# Structural basis for chemokine recognition and receptor activation of chemokine receptor CCR5

**DOI:** 10.1038/s41467-021-24438-5

**Published:** 2021-07-06

**Authors:** Hui Zhang, Kun Chen, Qiuxiang Tan, Qiang Shao, Shuo Han, Chenhui Zhang, Cuiying Yi, Xiaojing Chu, Ya Zhu, Yechun Xu, Qiang Zhao, Beili Wu

**Affiliations:** 1 CAS Key Laboratory of Receptor Research, Shanghai Institute of Materia Medica, Chinese Academy of Sciences, Shanghai, China; 2 State Key Laboratory of Drug Research, Shanghai Institute of Materia Medica, Chinese Academy of Sciences, Shanghai, China; 3 University of Chinese Academy of Sciences, Beijing, China; 4 School of Pharmaceutical Science and Technology, Hangzhou Institute for Advanced Study, University of Chinese Academy of Sciences, Hangzhou, China; 5 Zhongshan Branch, the Institute of Drug Discovery and Development, CAS, Zhongshan, China; 6 School of Life Science and Technology, ShanghaiTech University, Shanghai, China

**Keywords:** G protein-coupled receptors, Cryoelectron microscopy, X-ray crystallography

## Abstract

The chemokine receptor CCR5 plays a vital role in immune surveillance and inflammation. However, molecular details that govern its endogenous chemokine recognition and receptor activation remain elusive. Here we report three cryo-electron microscopy structures of G_i1_ protein-coupled CCR5 in a ligand-free state and in complex with the chemokine MIP-1α or RANTES, as well as the crystal structure of MIP-1α-bound CCR5. These structures reveal distinct binding modes of the two chemokines and a specific accommodate pattern of the chemokine for the distal N terminus of CCR5. Together with functional data, the structures demonstrate that chemokine-induced rearrangement of toggle switch and plasticity of the receptor extracellular region are critical for receptor activation, while a conserved tryptophan residue in helix II acts as a trigger of receptor constitutive activation.

## Abstract (web summary)

The chemokine receptor CCR5 plays multiple roles in the immune system. Here, structures of G_i1_ protein-coupled CCR5 with or without a chemokine bound and of the CCR5- chemokine MIP-1 α complex offer insight into the distinct binding modes of the ligands and into the mechanism of CCR5 activation.

## Introduction

In response to chemokines, a family of small proteins (8–10 kDa), chemokine receptors (CKRs) are essential for coordinating cell migration and positioning and are involved in many infectious and inflammatory diseases as well as tumor formation and metastasis^1^. The chemokine system has emerged as a complex network with 23 CKRs recognizing 50 chemokines in human cells^2^. The recognition between the CKRs and chemokines exhibits diversity and promiscuity, with one CKR binding to numerous chemokines while one chemokine recognizing multiple CKRs, which result in biased signaling and functional selectivity^3,4^. In addition, it has been found that some CKRs, including CCR5, can constitutively activate downstream signaling pathways in absence of agonist^5–7^, adding the complexity of function modulation in this receptor family. Recently, structures of several CKRs in complex with chemokine agonists or chemokine antagonists were determined using crystallography or cryo-electron microscopy (cryo-EM)^8–12^. These structures provide insights into chemokine binding and CKR activation, but molecular factors that govern the recognition between a CKR and different chemokines as well as CKR constitutive activation are unknown.

CCR5 is a potential drug target for a broad range of immune diseases^13^ and has been shown to be essential for human immunodeficiency virus (HIV) pathogenesis by acting as the principal coreceptor of the viruses^14^. CCR5 exerts its physiological functions by binding to multiple chemokines, such as macrophage inflammatory protein (MIP-1α, also known as CCL3) and RANTES (regulated on activation normal T cell expressed and secreted, also known as CCL5)^15^. Previous efforts on CCR5 structural studies enabled the determination of two inactive crystal structures of CCR5 bound to the marketed anti-HIV drug maraviroc^16^ and an antagonist variant of RANTES, [5P7]RANTES^10^. However, molecular mechanisms underlying endogenous chemokine ligand-binding and receptor activation of CCR5 stay ambiguous.

In this work, we report three cryo-EM structures of CCR5 bound to heterotrimeric G_i1_ protein in both ligand-free and chemokine (MIP-1α or RANTES)-bound states as well as the crystal structure of CCR5 in complex with MIP-1α. These structures, combined with data of cell signaling, crosslinking, and molecular dynamics (MD) simulations, provide molecular details that define recognition of various chemokines, and offer important insights into chemokine-induced and constitutive activation of CCR5.

## Results and discussion

### Structure determination of CCR5–chemokine and CCR5–G_i_ complexes

**Fig. 1:**
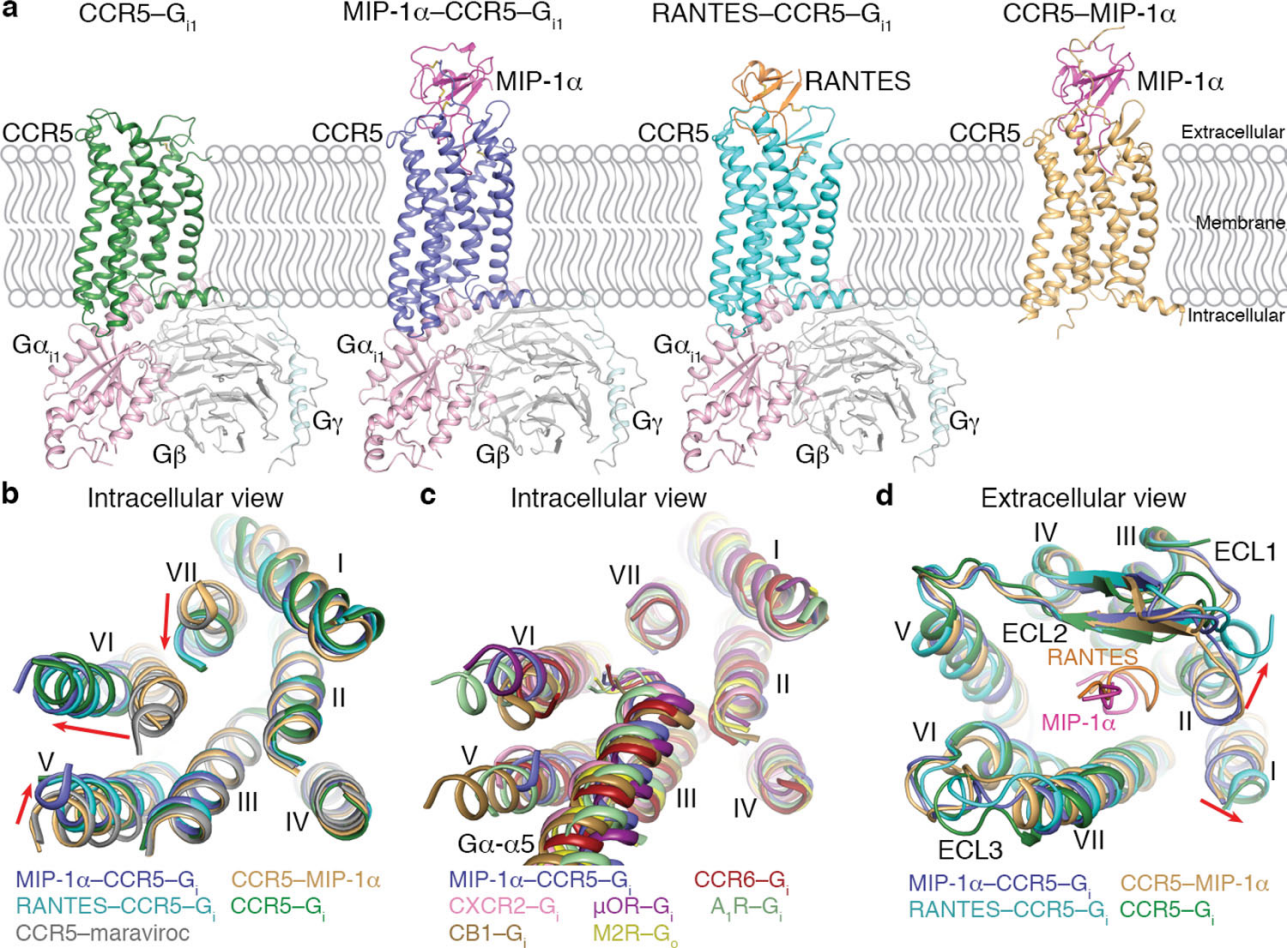
Overall structures of the CCR5–chemokine and CCR5–G_i1_ complexes. **a** Cryo-EM structures of CCR5–G_i1_, MIP-1α–CCR5–G_i1_, and RANTES–CCR5–G_i1_ and crystal structure of CCR5–MIP-1α. The structures are shown in cartoon representation. The receptor CCR5 in the four structures is colored green, blue, cyan, and gold, respectively. The chemokines MIP-1α and RANTES are colored magenta and orange, respectively. The three subunits in G_i1_ are colored light pink, light gray, and light cyan, respectively. Disulfide bonds are shown as yellow sticks. **b** Comparison of the transmembrane helical bundle conformation in the CCR5 structures. The helical bundles in the structures of CCR5–G_i1_, MIP-1α–CCR5–G_i1_, RANTES–CCR5–G_i1_, and CCR5–MIP-1α and the previously determined structure of CCR5–maraviroc (PDB ID: 4MBS) are colored green, blue, cyan, gold, and gray, respectively. The red arrows indicate the movements of the intracellular tips of helices V, VI, and VII in the G_i1_-bound CCR5 structures compared to those in the structures of CCR5–MIP-1α and CCR5–maraviroc. **c** Comparison of the G protein-binding pocket in the structures of MIP-1α–CCR5–G_i1_ and other class A GPCR–G_i/o_ complexes. The helical bundles and the C termini of Gα α5-helix in the MIP-1α–CCR5–G_i1_ structure and the structures of CCR6–G_i_, CXCR2–G_i_, μOR–G_i_, A_1_R–G_i_, CB1–G_i_, and M2R–G_o_ (PDB IDs: 6WWZ, 6LFO, 6DDE, 6D9H, 6N4B, and 6OIK) are colored blue, dark red, pink, purple, light green, brown, and yellow, respectively. **d** Comparison of the extracellular regions in the structures of CCR5–G_i1_, MIP-1α–CCR5–G_i1_, RANTES–CCR5–G_i1_, and CCR5–MIP-1α. The N-terminal regions in MIP-1α and RANTES (residues 1–9) are shown in cartoon representation and colored magenta and orange, respectively. The red arrows indicate movements of the extracellular tips of helices I and II in the RANTES–CCR5–G_i1_ structure compared to those in the MIP-1α-bound structures.

For structure determination of the ligand-free CCR5–G_i1_ complex, a mutation G163^4.60^N [superscript indicates Ballesteros–Weinstein nomenclature^17^] was introduced in the wild-type CCR5 and 33 residues (F320-L352) were truncated at the receptor C terminus to improve protein yield and homogeneity as previously reported^16^ (Supplementary Fig. 1a, b). The effect of these modifications on receptor function was assessed by a cAMP inhibition assay and an inositol phosphate (IP) accumulation assay using a chimeric Gα protein Gα_Δ6qi4myr_, which converts G_i_-related signaling into a G_q_ readout^18^. The data showed a wild-type level of basal and chemokine-induced activities for the engineered receptor (Supplementary Fig. 1c–g). The ligand-free CCR5–G_i1_ structure was determined by cryo-EM single-particle analysis with an overall resolution of 2.8 Å (Fig. 1a; Table 1; Supplementary Figs. 2a–f and 3a, b).

**Table 1:** Cryo-EM data collection, refinement, and validation statistics.

	CCR5–G_i1_ (EMDB-31424) (PDB 7F1S)	MIP-1α–CCR5–G_i1_ (EMDB-31422) (PDB 7F1Q)	RANTES–CCR5–G_i1_ (EMDB-31423) (PDB 7F1R)
***Data collection and processing***			
Magnification	81,000	81,000	81,000
Voltage (kV)	300	300	300
Electron exposure (e^–^/Å^2^)	70	70	70
Defocus range (μm)	–0.8 to –1.5	–0.8 to –1.5	–0.8 to –1.5
Pixel size (Å)	1.045	1.045	1.045
Symmetry imposed	C1	C1	C1
Initial particle images (no.)	5,511,331	7,095,732	6,624,475
Final particle images (no.)	1,531,142	745,096	1,304,062
Map resolution (Å)	2.8	2.9	3.0
FSC threshold	0.143	0.143	0.143
Map resolution range (Å)	2.5−5.0	2.5−5.0	2.5−5.0
***Refinement***			
Initial model used (PDB code)	6DDE, 4MBS	6DDE, CCR5–MIP-1α^a^	6DDE, 5UIW
Model resolution (Å)	2.8	2.9	3.0
FSC threshold	0.5	0.5	0.5
Map sharpening *B* factor (Å^2^)	–60	–85	–100
***Model composition***			
Receptor residues	276 (2,051 atoms)	298 (2,351 atoms)	277 (2,124 atoms)
Chemokine residues	/	69 (513 atoms)	50 (326 atoms)
G_i1_ residues	571 (4,162 atoms)	571 (4,266 atoms)	567 (4,171 atoms)
***B-factors (Å***^***2***^***)***			
Receptor	134.4	59.3	73.7
Chemokine	/	78.9	116.9
G_i1_	88.2	53.2	47.8
***R.m.s. deviations***			
Bond lengths (Å)	0.002	0.002	0.002
Bond angles (°)	0.516	0.521	0.469
**Validation**			
MolProbity score	1.63	1.65	1.41
Clash score	9.14	10.37	7.38
Poor rotamers (%)	0.17	0.00	0.15
***Ramachandran plot***			
Favored (%)	97.24	97.40	98.51
Allowed (%)	2.76	2.60	1.49
Disallowed (%)	0.00	0.00	0.00

^a^The crystal structure solved in this study.

To facilitate the formation of the CCR5–chemokine complexes and improve complex stability, in addition to the mutation and truncation, the chemokine C terminus and the N terminus of CCR5 were connected by a 12 × Gly-Ser linker, which resulted in high protein yield and homogeneity (Supplementary Fig. 1a, h, i). Additionally, a disulfide bridge between the receptor and chemokine [T16C (CCR5)-T15C (MIP-1α), E172C (CCR5)-F28C (RANTES)] was introduced to further facilitate structure determination (Supplementary Figs. 1a, j, k, 4a, and 5a, b; see Supplementary Note for disulfide design). Functional studies confirmed that both the GS linker and cysteine mutations had little effect on receptor signaling (Supplementary Fig. 1e, f). Using the optimized CCR5–chemokine proteins, the cryo-EM structures of MIP-1α–CCR5–G_i1_ and RANTES–CCR5–G_i1_ were determined with an overall resolution of 2.9 and 3.0 Å, respectively (Fig. 1a; Table 1; Supplementary Fig. 2g–r). The maps allowed unambiguous placement of most of residues in the receptor, chemokines, and G protein (Supplementary Fig. 3c–f), except for the N- and C-termini of CCR5 (residues 1-15 and 314-319 in the MIP-1α-bound CCR5; residues 1-32 and 314-319 in the RANTES-bound CCR5) and the N-loop and 40s-loop of RANTES (residues 15–24 and 42–46).

To obtain molecular details of CCR5-chemokine recognition at high resolution, crystallography studies of the CCR5–MIP-1α complex were also carried out. The complex protein was further optimized by introducing another six CCR5 mutations and inserting a rubredoxin fusion protein^19^ in the third intracellular loop (ICL3) of the receptor to improve the receptor stability and facilitate crystallization as suggested by previous structural studies of CCR5 and CCR9^16,20^ (Supplementary Fig. 1a, l). Our functional data revealed limited effects of these mutations on receptor signaling with a wild-type level of MIP-1α potency (EC_50_) and 70–110% of maximum response (span), except for the mutation A233^6.33^D that forms a salt bridge with the intracellular tip of helix I to limit the conformational change of helix VI (Supplementary Fig. 1m and Supplementary Table 1). The crystal structure of CCR5–MIP-1α was solved at 2.6 Å resolution (Fig. 1a; Table 2; Supplementary Fig. 3g, h).

**Table 2:** X-ray data collection and refinement statistics of CCR5–MIP-1α complex structure.

	CCR5–MIP-1α
***Data collection***^a^	
Space group	*C2*_*1*_
***Cell dimensions***	
*a, b, c* (Å)	49.1, 204.4, 69.0
α, β, γ (°)	90.0, 105.5, 90.0
Resolution (Å)	50.0–2.60 (2.64–2.60)^b^
*R*_*merge*_ (%)	11.2 (94.2)
*I / σ(I)*	31.7 (1.3)
*CC*_1/2_ (%)	98.5 (80.8)
Completeness (%)	94.9 (75.6)
Redundancy	6.7 (5.7)
***Refinement***	
Resolution (Å)	50.0–2.60
No. reflections	17,242 (888)
*R*_*work*_ */ R*_*free*_ (%)	22.8/27.1
***No. atoms***	
Protein	3,375
Zn	1
***Average B-factors (Å***^***2***^***)***	
CCR5	111.1
MIP-1α	119.8
Rubredoxin	153.2
Zn	225.4
***R.m.s. deviations***	
Bond lengths (Å)	0.011
Bond angles (°)	1.604
***Ramachandran plot***	
Favored (%)	95.67
Allowed (%)	4.33
Disallowed (%)	0.00

^a^Data from 40 crystals were used to solve the structure.

^b^Numbers in parentheses refer to the highest-resolution shell.

### Overall architecture of CCR5 bound to chemokine and/or G_i1_

In absence of G protein, the CCR5–MIP-1α crystal structure adopts an inactive conformation with its helix VI in an inward position, a state similar to that in our previously determined inactive structure of CCR5 bound to the inverse agonist maraviroc^16^ (Fig. 1b). In contrast, the complexes of MIP-1α–CCR5–G_i1_, RANTES–CCR5–G_i1_, and ligand-free CCR5–G_i1_ are in an alike active conformation despite different chemokine-bound states, with the receptor Cα (residues L33-Q313) root-mean-square deviation (RMSD) of 1.4 Å (MIP-1α–CCR5–G_i1_ vs. RANTES–CCR5–G_i1_) and 1.7 Å (MIP-1α–CCR5–G_i1_ vs. CCR5–G_i1_) (Fig. 1a). The intracellular region of the receptor adopts a similar conformation in the three G_i1_-bound CCR5 structures (Fig. 1b). To accommodate the G protein, helix VI moves outwards by approximate 10 Å and helices V and VII shift inwards by about 3 and 5 Å, respectively, compared to those in the inactive CCR5 structure (Fig. 1b). Structural comparison of the G_i1_-bound CCR5 structures and other known class A GPCR–G_i/o_ structures revealed a similar G protein-binding cavity on the receptor intracellular surface, where the backbone conformations overlay for both the receptor and the C terminus of Gα α5-helix (Fig. 1c).

In contrast to the similarity on the receptor intracellular side, structural deviation occurs in the extracellular loops and the extracellular regions of helices I and II in CCR5 (Fig. 1d). The extracellular ends of helices I and II move away from the central axis of the transmembrane helical bundle by about 5 Å in the RANTES–CCR5–G_i1_ structure relative to those in the MIP-1α-bound structures. This alteration is most likely associated with the different binding modes of the chemokine N terminus, in which the residues Y3–D6 of RANTES are adjacent to the extracellular tip of helix II while the counterpart in MIP-1α shifts towards helix VII (Fig. 1d). Lacking interaction with the chemokine, the ligand-free CCR5–G_i1_ structure exhibits a more open ligand-binding pocket compared with the chemokine-bound CCR5 structures due to the outward shift of helix I, the second extracellular loop (ECL2), and the third extracellular loop (ECL3) (Supplementary Fig. 5c). These differences suggest the involvement of the receptor extracellular region in modulating chemokine binding.

**Fig. 2:**
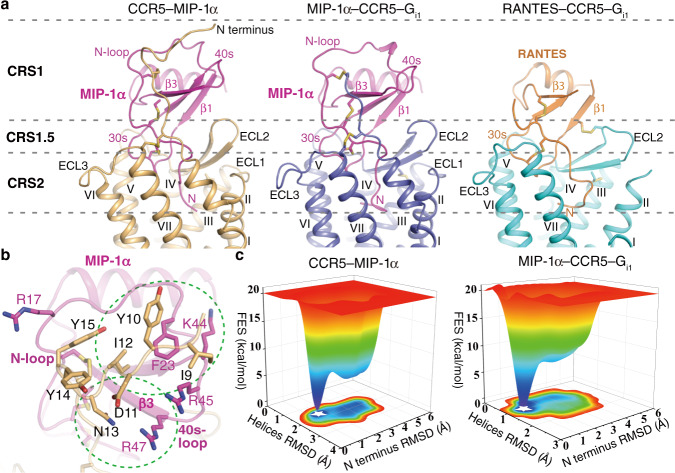
Overall chemokine binding mode in CCR5 and receptor-chemokine recognition in CRS1. **a** Overall chemokine binding mode in CCR5. The structures of CCR5–MIP-1α, MIP-1α–CCR5–G_i1_, and RANTES–CCR5–G_i1_ are shown in cartoon representation. The receptors in the three structures are colored gold, blue, and cyan, respectively. The chemokines MIP-1α and RANTES are colored magenta and orange, respectively. The regions of CRS1, CRS1.5, and CRS2 are indicated by gray dashed lines. **b** The receptor-chemokine interactions in the CRS1 of the CCR5–MIP-1α structure. The key residues of CCR5 and MIP-1α that form contacts are shown as sticks with gold and magenta carbons, respectively. The two interaction cores other than the interactions between the tyrosines in CCR5 and the basic residues in MIP-1α are indicated by green dashed circles. **c** The free energy surface (FES) of the GaMD simulations of the CCR5–MIP-1α and MIP-1α–CCR5–G_i1_ complexes. The FES profile was calculated along with the collective variables of the RMSDs of the CCR5 helices and N terminus. The experimentally identified binding states between MIP-1α and the CCR5 N terminus are highlighted with white-colored stars. Source data are provided as a Source Data file.

Similar to the previously determined structure of CCR5 in complex with the chemokine antagonist [5P7]RANTES^10^, the MIP-1α- and RANTES-bound CCR5 structures reveal three receptor-chemokine interaction epitopes: (1) chemokine recognition site 1 (CRS1), in which the receptor N terminus (residues P8-E18) occupies a shallow groove shaped by the N-loop, 40s-loop, and β3-strand of the chemokine; (2) CRS1.5, where the CCR5 residues P19-K22 form an anti-parallel β-sheet with the chemokine residues T8-C11; and (3) CRS2, where the N terminus, β1-strand, and 30s-loop of the chemokines penetrate into the ligand-binding pocket within the transmembrane helical bundle, forming contacts with ECL2, ECL3, and helices I, II, III, V, VI, and VII (Fig. 2a). The CCR5–chemokine complexes exhibit a deep ligand-binding pocket, which is similar to that in the previously determined structures of CXCR4–vMIP-II and US28–CX3CL1 complexes, but with more extensive receptor-chemokine interactions compared to those in the known chemokine-bound structures of other CKRs (Supplementary Fig. 6a). The calculated binding interface between the chemokine and the transmembrane helices and ECLs of the receptor is about 300 Å^2^ larger for MIP-1α and RANTES in CCR5 (1,190 and 1,029 Å^2^) than that for vMIP-II in CXCR4^9^, CX3CL1 in US28^8^, CCL20 in CCR6^11^, and CXCL8 in CXCR2^12^ (806, 748, 759, and 836 Å^2^, respectively). Furthermore, different chemokines adopt distinct orientations when bound to their respective receptors (Supplementary Fig. 6b). These structural differences highlight the complexity and diversity of the chemokine recognition mechanism of CKRs.

### Chemokine recognition in CRS1

The N terminus of CKRs has been suggested to be pivotal for chemokine recognition^21^. However, molecular mechanism of receptor-chemokine recognition in CRS1 remains elusive due to the lack of an intact receptor N terminus in the previously determined CKR–chemokine complex structures. Our crystal structure of the CCR5–MIP-1α complex allowed observation of interactions between the chemokine and the distal N-terminal segment till residue P8 in CCR5, providing insights into the chemokine-receptor recognition. Similar to the binding mode previously proposed by modeling and NMR studies of CCR5^10,22^, the receptor N terminus (residues P8–E18) binds to a cleft between the N-loop and the 40s-loop and β3-strand in MIP-1α (Fig. 2b). In the CCR5–MIP-1α structure three tyrosine residues in the receptor N terminus, Y10, Y14, and Y15, which are potentially sulfated and have been suggested to be essential for binding to positively charged residues on the binding surface of chemokines^10,22,23^, are surrounded by four basic residues in MIP-1α including R17, K44, R45, and R47, with Y10 adjacent to K44 and R45 while Y14 and Y15 approaching R17 and R47 (Fig. 2b). Although no polar interaction between these residues was observed due to lack of sulfation, this binding mode may represent a low-affinity binding state between the CCR5 N terminus and MIP-1α, as previous NMR studies using peptides corresponding to the N termini of CKRs indicated that nonsulfated peptides bound to the same region as the sulfopeptides, albeit more weakly^23^. To further explore the recognition pattern of the tyrosine residues, we carried out disulfide crosslinking studies by co-expressing cysteine mutants of CCR5 and MIP-1α in HEK293F cells with tyrosylprotein sulfotransferase 1 (TPST-1) co-transfected to facilitate tyrosine sulfation^24,25^. Complex formation was shown when the CCR5 mutant Y10C was co-expressed with the MIP-1α mutant K44C or R45C as well as when Y14C or Y15C (CCR5) was co-expressed with R17C or R47C (MIP-1α) (Supplementary Fig. 4b), consistent with the positioning of these residues observed in the CCR5–MIP-1α structure. Furthermore, the CCR5 mutant Y10C was also crosslinked with the MIP-1α mutant R17C or R47C (Supplementary Fig. 4b), suggesting that the receptor N terminus retains a degree of conformational dynamics when bound to the chemokine.

In addition to the above tyrosine-based interactions, the CCR5–MIP-1α crystal structure reveals other two interaction cores between the receptor and chemokine, including a hydrophobic interaction network formed between the residues I9, Y10, and I12 in CCR5 and the MIP-1α residue F23 as well as a polar interaction core composed of the chemokine residue R47 and the receptor residues D11 and N13 (Fig. 2b). These close contacts were also verified by crosslinking, showing the successful formation of trapped CCR5–MIP-1α complex when the chemokine mutant F23C or R47C was co-expressed with the mutant of either of its binding partners in the receptor (Supplementary Fig. 4c). Taken together, the crosslinking data support that the groove built by the N-loop, 40s-loop, and β3-strand of MIP-1α is the recognition site for the N terminus of CCR5.

Additionally, to exclude the possible effect of crystal packing on receptor-chemokine interaction in CRS1, we performed three independent 1 μs MD simulations on the CCR5–MIP-1α crystal structure with the ICL3-rubredoxin fusion and all mutations removed. In parallel, three independent MD simulations on the cryo-EM structure of MIP-1α–CCR5–G_i1_, where the missing N-terminal residues of CCR5 (P8-Y15) were added according to the conformation of the counterpart in the crystal structure, were carried out for comparison. To achieve extensive conformational sampling and thus quantitatively estimate the thermodynamically weighted conformational ensembles, the Gaussian accelerated molecular dynamics (GaMD), a sophisticated enhanced sampling MD method^26,27^, instead of classical unbiased MD was used.

The simulation data show that CCR5 is more structurally dynamic in the absence of G_i1_ coupling, as its RMSD and root-mean-square fluctuation (RMSF) values are generally larger in the trajectories of CCR5–MIP-1α compared to those of MIP-1α–CCR5–G_i1_ (Supplementary Fig. 7a, b). The structural dynamics of MIP-1α, on the other hand, is barely influenced by the G_i1_ coupling except at its N terminus (Supplementary Fig. 7b). In this context, the G_i1_ coupling seems to stabilize the conformation of the receptor which, in turn, exerts an indirect constraining effect on the structural dynamics of the chemokine N terminus that deeply inserts into the receptor. Such an effect, however, could not extend to the remaining part of the chemokine, which is packed out of the binding cavity within the receptor helical bundle. Additionally, the simulations demonstrate that although the receptor N terminus is capable of visiting a large conformational space, its energetically favorable state is always close to the one seen in the crystal structure of CCR5–MIP-1α that contacts the cleft between the N-loop and the 40s-loop and β3-strand in MIP-1α (Fig. 2c). The G_i1_ coupling further reduces the tendency of the receptor N terminus to explore other conformations than the experimentally identified one. In line with this, almost all of the receptor and chemokine amino acid pairs studied in the disulfide crosslinking experiments (Supplementary Fig. 4b, c), except Y15-R47, have the chance to be in close proximity to each other in the simulations (Supplementary Fig. 7c).

In the previously proposed two-step model for chemokine binding and CKR activation, the initial binding interaction of chemokine occurs in the N-terminal tail of the receptor^28^, suggesting that the receptor-chemokine interactions in CRS1 play a crucial role in chemokine ligand selectivity. Indeed, comparison of the CCR5–MIP-1α structure and the recently published structure of CXCL8–CXCR2–G_i_^12^ revealed distinct binding modes for the receptor N terminus. Unlike the CCR5 structure, where the receptor N terminus (residues P8–Y15) runs in parallel with the β3-strand of the chemokine, the N-terminal segment S26–L33 in CXCR2 extends across the N-loop and approaches the C-terminal α-helix of CXCL8 (Supplementary Fig. 6c). This difference reflects the diversity of the receptor-chemokine recognition pattern in CRS1, which may result from distinct natures of the receptor N termini such as electrostatic distribution and corresponding recognition requirement for the binding cleft in the chemokines^9^.

### Distinct interaction patterns of MIP-1α and RANTES in CRS2

**Fig. 3:**
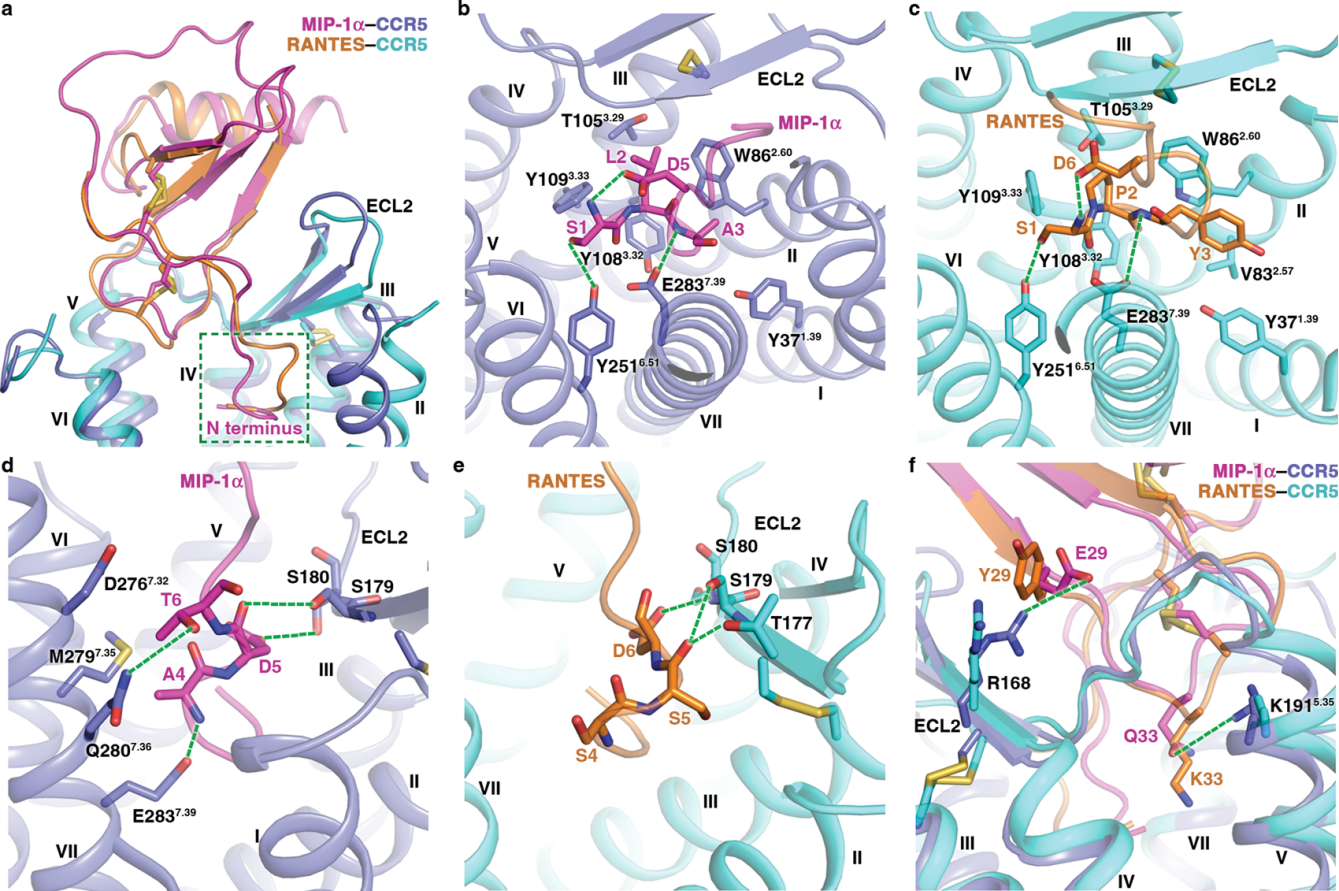
Binding modes of MIP-1α and RANTES in CRS2. **a** Comparison of the binding poses of MIP-1α and RANTES in CCR5. The structures of MIP-1α–CCR5–G_i1_ and RANTES–CCR5–G_i1_ are shown in cartoon representation and colored blue (CCR5)/magenta (MIP-1α) and cyan (CCR5)/orange (RANTES). The N-terminal regions of the chemokines are highlighted by a green dashed box. **b**, **d** Binding mode of the N-terminal residues of MIP-1α. **b** Binding mode of the MIP-1α residues S1–A3; **d** binding mode of the MIP-1α residues A4-T6. The MIP-1α–CCR5–G_i1_ structure is shown in cartoon representation. The MIP-1α and CCR5 residues that are involved in interactions are shown as sticks with magenta and blue carbons, respectively. The polar interactions are displayed as green dashed lines. **c**, **e** Binding mode of the N-terminal residues of RANTES. **c** Binding mode of the RANTES residues S1–Y3; **e** binding mode of the RANTES residues S4-D6. The RANTES–CCR5–G_i1_ structure is shown in cartoon representation. The RANTES and CCR5 residues that are involved in interactions are shown as sticks with orange and cyan carbons, respectively. The polar interactions are displayed as green dashed lines. **f** Comparison of interactions formed by the chemokine residues 29 and 33. The structures of MIP-1α–CCR5–G_i1_ and RANTES–CCR5–G_i1_ are shown in cartoon representation. The MIP-1α residues E29 and Q33 and the CCR5 residues R168 and K191^5.35^ form polar interactions in the MIP-1α–CCR5–G_i1_ structure and the counterparts in the RANTES–CCR5–G_i1_ structure are shown as sticks.

Both MIP-1α and RANTES bind to CCR5 with high affinities^29–31^. These two chemokines share 49% amino acid identity in the core domain (C10-C terminus) but display high diversity in the N-terminal segment proceeding the first two cysteines (residues 1–9). This agrees with the MIP-1α- and RANTES-bound CCR5 structures, in which the two chemokines show distinct binding modes in their N-terminal regions, where the residues occupy different binding sites within the receptor ligand-binding pocket, and align well in the rest of the ligands (Fig. 3a). In the structures of MIP-1α–CCR5–G_i1_ and RANTES–CCR5–G_i1_, the N termini of the chemokines adopt a ‘hook’-like conformation, which is stabilized by a salt bridge between the only negatively charged residue in this region (D5 in MIP-1α, D6 in RANTES) and the positively charged main-chain nitrogen of residue S1 (Fig. 3a–c). The first three residues of the chemokines sit at bottom of the ligand-binding pocket, packing against helices II and III of the receptor and forming hydrophobic contacts with W86^2.60^, T105^3.29^, Y108^3.32^, and F109^3.33^ (Fig. 3b, c). The importance of this interaction core in chemokine recognition is supported by previous mutagenesis studies of RANTES, showing an over 5,000-fold reduction of CCR5 binding affinity for the alanine replacement of the residue P2 (ref. ^32^), which plays a major role in mediating the interactions within the hydrophobic core. Additionally, in both chemokine–CCR5–G_i1_ complexes the side chain of the N-terminal residue S1 forms a hydrogen bond with the receptor residue Y251^6.51^ and the main chain of the third residue in the chemokines makes a hydrogen bond with E283^7.39^ (Fig. 3b, c). These interactions are consistent with the previously reported impairment of MIP-1α and RANTES binding associated with the mutations Y251^6.51^A and E283^7.39^A^33^. In the crystal structure of CCR5–MIP-1α, the N-terminal residues S1 and L2 in MIP-1α were not modeled due to poor electron densities, suggesting that the chemokine N terminus is more dynamic in the G protein-free state compared to that in the G_i1_-bound complex, supported by the larger RMSF values of the chemokine N terminus in the former structure than in the latter as revealed by the MD simulations (Supplementary Fig. 7b). This may correlate with the previous observation that coupling to the G protein is necessary for CCR5 to bind the chemokines with high affinity^34,35^.

The third residues in the two chemokines vary in their side chains with alanine in MIP-1α but a tyrosine in RANTES, resulting in different binding modes at this position. The bulky side chain of the RANTES residue Y3 wedges into a gap between helices I and II in CCR5 and makes contacts with Y37^1.39^, V83^2.57^, and W86^2.60^, leading to the outward movements of these two helices relative to those in the MIP-1α-bound structures (Figs. 1d and 3c). This conformational difference is also associated with the different binding poses of residues 4–6 in the two chemokines. Accompanying the outward shift of helix II in the RANTES-bound CCR5 structure, the chemokine residues S4–D6 occupy a binding site adjacent to helix II and ECL2 and form polar interactions with residues T177, S179, and S180 in ECL2 (Fig. 3e). In contrast, the MIP-1α residues A4–T6 approach helix VII due to a spatial hindrance caused by helix II, with their conformation stabilized by interacting with D276^7.32^, M279^7.35^, Q280^7.36^, and E283^7.39^ (Fig. 3d).

In addition to the differences in the N-terminal region of the chemokines, the receptor-chemokine interactions also differ in some regions within the core domain owing to residue variations between the two chemokines. For instance, the MIP-1α residue E29 forms a salt bridge with R168 in the CCR5 ECL2, while the counterpart in RANTES, Y29, disrupts the ionic interaction with the receptor (Fig. 3f). Similarly, residue Q33 in MIP-1α makes a hydrogen bond with residue K191^5.35^ at the extracellular end of helix V, which repels the positively charged K33 in RANTES (Fig. 3f). Consistent with these differences, previous mutagenesis studies of CCR5 showed that the mutants R168A and K191^5.35^A diminished binding with MIP-1α but had little impact on RANTES binding^31,36^.

### Chemokine-induced activation of CCR5

It has been suggested that the core region of the chemokines contributes most of the binding energy while the chemokine N terminus is required for functional activation of CCR5, as the N-terminal truncated chemokines could still bind to the receptor but could not trigger receptor signaling^37,38^. Furthermore, the previous characterization of RANTES showed that the alanine substitutions of most of the N-terminal residues (S1–T8) induced a calcium flux response of CCR5 that was 40–70% of the response to the wild-type RANTES with little change in binding affinity^32^. These observations indicate that the interactions between the receptor and chemokine N terminus play a crucial role in receptor activation. This is supported by our IP accumulation assay, showing that mutations of the CCR5 residues Y37^1.39^, W86^2.60^, T105^3.29^, Y108^3.32^, F109^3.33^, Y251^6.51^, and E283^7.39^, which establish an interaction network with the chemokine N terminus in the two chemokine–CCR5–G_i1_ structures (Fig. 3b, c), substantially impaired potency and/or maximal response of MIP-1α and RANTES (Supplementary Fig. 8 and Supplementary Table 1).

**Fig. 4:**
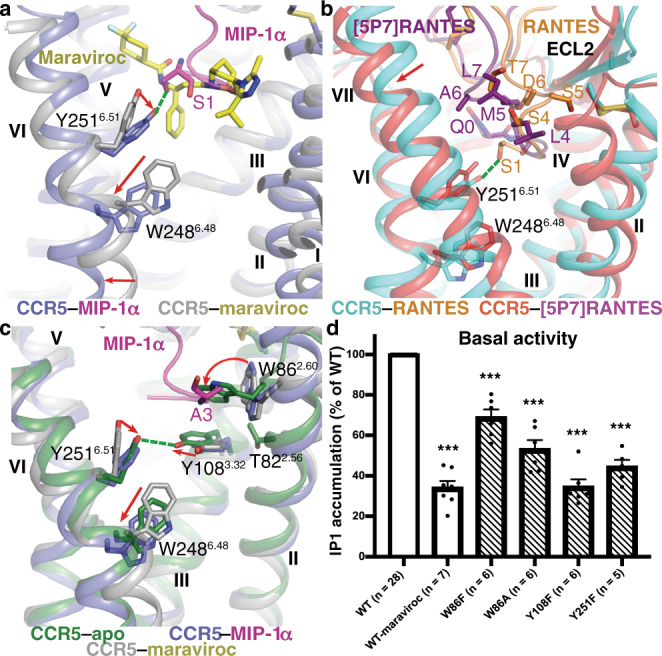
Chemokine-induced and constitutive activation of CCR5. **a** Chemokine-induced conformational change of W248^6.48^ and Y251^6.51^. The structure of MIP-1α–CCR5–G_i1_ is shown in cartoon representation and colored blue (CCR5) and magenta (MIP-1α). The CCR5–maraviroc structure is shown in cartoon representation and colored gray. The ligand maraviroc is shown as yellow sticks. The receptor residues W248^6.48^ and Y251^6.51^ in the two structures and the MIP-1α residue S1 are shown as sticks. The hydrogen bond between Y251^6.51^ and S1 in the MIP-1α–CCR5–G_i1_ structure is displayed as a green dashed line. The red arrows indicate the conformational changes of W248^6.48^ and Y251^6.51^ and the outward movement of helix VI in the MIP-1α–CCR5–G_i1_ structure relative to those in the CCR5–maraviroc structure. **b** Structural comparison of the RANTES–CCR5–G_i1_ and CCR5–[5P7]RANTES (PDB ID: 5UIW) complexes. The receptors in the two structures are colored cyan and red, respectively. The chemokines RANTES and [5P7]RANTES are colored orange and purple, respectively. The RANTES residues S1 and S4–T7 and the [5P7]RANTES residues Q0 and L4–L7 are shown as sticks. The hydrogen bond between Y251^6.51^ and S1 in the RANTES–CCR5–G_i1_ structure is displayed as a green dashed line. The red arrow indicates the movement of the extracellular end of helix VII in the CCR5–[5P7]RANTES structure compared to that in the RANTES–CCR5–G_i1_ structure. **c** Structural comparison of the CCR5–G_i1_ (CCR5-apo), MIP-1α–CCR5–G_i1_, and CCR5–maraviroc complexes. The receptors in the three structures are colored green, blue, and gray, respectively. MIP-1α is colored magenta. The receptor residues W86^2.60^, Y108^3.32^, W248^6.48^, and Y251^6.51^ as well as the MIP-1α residue A3 are shown as sticks. The red arrows indicate the conformational changes of the four receptor residues in the CCR5–G_i1_ structure relative to those in the inactive CCR5–maraviroc structure. The residue T82^2.56^ in the CCR5–G_i1_ structure is also displayed as sticks, showing close proximity to W86^2.60^ and Y108^3.32^. **d** Basal activity of the wild-type CCR5 (WT) and mutants of residues involved in constitutive activation. The IP accumulation assay was performed in parallel with the measurement of the IP production using the cells only transfected with the chimeric Gα protein Gα_Δ6qi4myr_ as a control. The basal activity was calculated by subtracting the IP production measured in the control for the WT receptor and all the mutants and is shown as per cent of the WT activity. The numbers of independent experiments (*n*) performed in triplicate for the WT and mutants are shown in the parentheses. ***P < 0.0001 by one-way ANOVA followed by Dunnett’s post-test, compared with the basal activity of the WT (WT-maraviroc, W86F, W86A, Y108F, and Y251F: *P* < 0.0001). See Supplementary Table 1 for detailed statistical evaluation and expression level. Source data are provided as a Source Data file.

Induced by the interaction with the N-terminal residue S1 of the chemokines, the side chain of Y251^6.51^ shifts downwards in the chemokine-bound active structures relative to that in the inactive structure of CCR5–maraviroc (Fig. 4a). This movement results in a rotamer conformational change of W248^6.48^, which is known as the ‘toggle switch’ of class A GPCRs^39^, subsequently leading to the outward rearrangement of helix VI on the intracellular side to accommodate the G protein (Fig. 4a). The inverse agonist maraviroc, which occupies a binding site similar to that of the chemokine N terminus, blocks the conformational change of Y251^6.51^ by forming a spatial hindrance and makes close contact with W248^6.48^ to stabilize its inactive conformation (Fig. 4a). In contrast to the direct alteration of the conformation of the conserved motif in helix VI, a more indirect effect of the chemokine binding was observed in the recently determined G protein-bound CCR6 and CXCR2 structures, in which their chemokine ligands bind to a shallow cavity on the receptor extracellular surface and activate the receptors by inducing a conformational change in the extracellular region^11,12^. These observations highlight the diversity of receptor activation mechanisms of CKRs.

Previous mutagenesis and MD simulation studies of CCR5 suggested that a conserved CKR motif T^2.56^xP^2.58^ in helix II was involved in the activation process of CCR5 with the mutation P84^2.58^A attenuating the functional response to the chemokines^29^. Although not making any direct contact with the chemokines as shown in the chemokine-bound CCR5 structures, the residue P84^2.58^ introduces a kink in helix II, which brings conformational flexibility in this region. Indeed, on the extracellular side of the transmembrane helical bundle helix II exhibits the most diverse conformations when comparing the three G_i1_-bound CCR5 structures (Fig. 1d), implying the importance of the structural plasticity of this region in chemokine recognition and receptor activation. As mentioned above, the different positioning of helix II is associated with the distinct binding modes of the N-terminal regions in MIP-1α and RANTES. Reducing the helix plasticity by abrogating the proline kink through the mutation P84^2.58^A would likely influence the chemokine binding ability of the receptor and furthermore, relocate the residue W86^2.60^ to disturb the interaction network between the chemokine N terminus and the bottom of the receptor ligand-binding pocket, resulting in impaired receptor function. This finding demonstrates the importance of the structural integrity of the receptor extracellular region in receptor activation.

Using a phage display strategy, a series of N-terminally modified RANTES variants that differ in capacity to induce CCR5 signaling were discovered^40^. It has been noticed that almost all agonist variants as well as RANTES contain polar residues in positions 4–7, whereas these residues are hydrophobic in the antagonists such as [5P7]RANTES^10,41^, suggesting that this region plays a role in modulating receptor activation. Comparison of the RANTES–CCR5–G_i1_ structure and the previously published crystal structure of CCR5–[5P7]RANTES revealed distinct binding poses of the chemokine residues 4–7 (Fig. 4b). The residues S4-T7 of RANTES mainly form a polar interaction core with the receptor ECL2, while the counterpart in [5P7]RANTES packs against helices I and VII. Owing to this different binding mode, the extracellular tip of helix VII moves towards helix VI in the CCR5–[5P7]RANTES complex relative to that in the RANTES-bound structure, potentially stabilizing the receptor in an inactive state by constraining the conformational rearrangement of helix VII (Fig. 4b). Furthermore, occupying a slightly higher binding site, the N terminus of [5P7]RANTES lacks any contact with the key residue Y251^6.51^, and thus, fails to induce the conformational change of the ‘toggle switch’ (Fig. 4b). In contrast, a recently reported cryo-EM structure of CCR5 in complex with the super-agonist [6P4]RANTES and G_i_ reveals a deeper binding site for the N terminus of the RANTES analog, which alters the conformation of W248^6.48^ and leads to the rearrangement of helix VI by inducing a relocation of residue M287^7.43^ (ref. ^42^). Taken together, these structural findings suggest multiple activation modes of CCR5 and support the central role of the chemokine N terminus being the signaling trigger.

### Constitutive activation of CCR5

There is increasing evidence supporting the ability of CCR5 constitutively activating G protein signaling pathways^5,6^. Our ligand-free CCR5–G_i1_ structure provides molecular details about how a CKR activates the G protein in the absence of an agonist. Close inspection of the structure revealed conformational rearrangements of the aromatic residues at the bottom of the ligand-binding cavity (Fig. 4c). Compared to the structures of the chemokine–CCR5–G_i1_ complexes and inactive CCR5, side chain of the conserved CKR residue W86^2.60^ rotates toward helix VII by approximate 90° in the apo structure, occupying a similar position to the binding site of the residue 3 in the chemokines. The movement of W86^2.60^, together with an inward shift of helix II, further pushes the residue Y108^3.32^ toward helix VI. To form a hydrogen bond with the side chain of Y108^3.32^, the residue Y251^6.51^ undergoes a downward shift (relative to that in the inactive CCR5 structure), placing its side chain in a conformation similar to that in the chemokine–CCR5–G_i1_ complexes. This rearrangement results in the conformational change of W248^6.48^ and subsequent rearrangement on the receptor intracellular side, leading to receptor activation. Nevertheless, this does not rule out the possibility that the specific conformations of these key residues on the extracellular side are stabilized by and/or resulted from the G protein coupling.

To verify the involvement of these aromatic residues in CCR5 constitutive activation, basal activities of their mutants and the wild-type receptor were measured using the IP accumulation assay. Consistent with the structural finding, the data show an impaired basal activity for all the mutants tested, which had cell surface expression levels comparable to the wild type (Fig. 4d and Supplementary Table 1). The mutant W86^2.60^F displayed a basal activity that is 70% of the wild-type response, while the alanine replacement further reduced the activity to 50%, supporting the critical role of this residue in inducing receptor constitutive activation by altering the interactions within the aromatic residue cluster through the conformational change of its bulky side chain. The residues Y108^3.32^ and Y251^6.51^ also showed a large effect with their phenylalanine substitutions decreasing the basal activity by 55–65%, a level similar to that of the wild-type CCR5 in presence of the inverse agonist maraviroc. This result demonstrates the importance of the hydrogen bond between the hydroxyl groups of these two tyrosines in triggering the constitutive activation. Previous mutagenesis studies identified a constitutively active CCR5 mutant with a proline substitution for T82^2.56^ in the conserved T^2.56^xP^2.58^ motif. This mutant exhibited high basal signaling that was not further increased by chemokines^43^. Such high constitutive activity may be achieved by further facilitating the conformational rearrangement of the neighboring residues W86^2.60^ and Y108^3.32^ through a reorientation of helix II (Fig. 4c).

Collectively, this work provides a full picture of CCR5 in both chemokine-induced and constitutive activation states and reveals distinct molecular details that define the binding of different chemokines. These results shed light on the complexity of chemokine recognition and receptor activation of CCR5, and thereby greatly expand our knowledge about signal transduction by chemokine receptors.

## Methods

### Cloning and protein expression

For cryo-EM studies, the human CCR5 gene was cloned into a modified pFastBac1 vector (Invitrogen) with a haemagglutinin (HA) signal peptide followed by a 2 × strep tag at the N terminus and a Flag tag at the C terminus. The C-terminal residues F320–L352 of CCR5 were truncated and a mutation G163^4.60^N was introduced to improve protein yield and homogeneity. To obtain stable CCR5–chemokine complexes, the wild-type genes of MIP-1α and RANTES were connected with the N terminus of CCR5, respectively, with a 12 × GS linker. A disulfide bond between the chemokine and receptor was further introduced by mutating the MIP-1α residue T15 and CCR5 residue T16 to cysteines or replacing the RANTES residue F28 and CCR5 residue E172 with cysteines. A dominant-negative Gα_i1_ (DNGα_i1_) gene was generated by introducing five mutations, S47C, G202T, G203A, E245A, and A326S (ref. ^44^), and then cloned into the pFastBac1 vector. The human Gβ_1_γ_2_ gene with a 6 × His tag at the N terminus of Gβ_1_ was cloned into the pFastBac Dual vector (Invitrogen). The modified CCR5, CCR5–MIP-1α, or CCR5–RANTES was co-expressed with DNGα_i1_ and Gβ_1_γ_2_ in HighFive insect cells (Invitrogen) using Bac-to-Bac Baculovirus Expression System (Invitrogen). The cells at a density of 1.5 × 10^6^ cells per ml were infected with high-titre viral stocks (> 10^9^ viral particles per ml) at a multiplicity of infection (MOI) ratio of 5: 4: 4 for CCR5/CCR5–chemokine, DNGα_i1_, and Gβ_1_γ_2_. Cells were grown at 27 °C for 48 h and then harvested by centrifugation and stored at –80 °C until use.

To solve the crystal structure of CCR5–MIP-1α, the modified CCR5–MIP-1α gene was further optimized by introducing another six mutations including C58^1.60^Y, M64^2.38^A, A233^6.33^D, R274^7.30^A, T284^7.40^A, and K303E. To facilitate crystallization, a rubredoxin fusion protein^19^ was inserted between R223 and E227 at the third intracellular loop (ICL3) of CCR5. The Bac-to-Bac Baculovirus Expression System (Invitrogen) was used to generate high-titre recombinant baculovirus. *Spodoptera frugiperda* (*Sf*9) cells (Invitrogen) at a density of 2–3 × 10^6^ cells per ml were infected with a virus at an MOI of 5. The cells were grown at 27 °C for 48 h and then harvested by centrifugation and stored at –80 °C until use.

### Purification of chemokine–CCR5–G_i1_ and CCR5–G_i1_ complexes

The cells expressing the MIP-1α–CCR5–G_i1_, RANTES–CCR5–G_i1_, or CCR5–G_i1_ complex were thawed on ice and suspended in a buffer containing 20 mM HEPES, pH 7.5, 50 mM NaCl, 2 mM MgCl_2_, and EDTA-free protease inhibitor cocktail tablets (Roche). Then the suspension was incubated at room temperature for 1 h after adding apyrase (25 mU ml^–1^). The protein was extracted by adding 1% (w/v) lauryl maltoseneopentyl glycol (LMNG, Anatrace) and 0.1% (w/v) cholesterol hemisuccinate (CHS, Sigma). The mixture was incubated at 4 °C for 3 h. The supernatant was isolated by centrifugation at 160,000 *g* for 30 min, and then incubated with Strep-Tactin Sepharose (IBA Lifesciences) overnight at 4 °C.

The resin was washed with 2 column volumes of a buffer containing 20 mM HEPES, pH 7.5, 100 mM NaCl, 2 mM MgCl_2_, 0.01% (w/v) LMNG, and 0.001% (w/v) CHS, and then incubated with a buffer containing 20 mM HEPES, pH 7.5, 100 mM NaCl, 2 mM MgCl_2_, 0.8% (w/v) LMNG, 0.27% (w/v) glyco-diosgenin (GDN, Anatrace), and 0.08% (w/v) CHS at 4 °C for 2 h. The resin was then washed with 20 column volumes of washing buffer containing 20 mM HEPES, pH 7.5, 100 mM NaCl, 2 mM MgCl_2_, 0.01% (w/v) LMNG, 0.0033% (w/v) GDN, and 0.001% (w/v) CHS. The complex was eluted with 5 column volumes of elute buffer containing 150 mM Tris-HCl, pH 8.0, 100 mM NaCl, 2 mM MgCl_2_, 50 mM Biotin, 0.01% (w/v) LMNG, 0.0033% (w/v) GDN, and 0.001% (w/v) CHS, and further purified by size-exclusion chromatography using a Superdex 200 Increase 10/300 column (GE Healthcare), which was pre-equilibrated with 20 mM HEPES, pH 7.5, 100 mM NaCl, 0.002% (w/v) LMNG, 0.00067% (w/v) GDN, and 0.0002% (w/v) CHS. The purified complex was concentrated to 3 mg ml^–1^ with a 100-kDa molecular weight cut-off concentrator (Millipore), and analyzed by SDS-PAGE and analytical size-exclusion chromatography.

### Cryo-EM data acquisition and processing

The G_i1_-bound complexes were diluted to 1.5 mg ml^–1^ using a buffer containing 20 mM HEPES, pH 7.5, 100 mM NaCl, 0.002% (w/v) LMNG, 0.00067% (w/v) GDN, and 0.0002% (w/v) CHS. Then 3 μl of protein sample was applied to glow-discharged holey grids (CryoMatrix R1.2/1.3, Au 300 mesh) and vitrified at 4 °C and 100% humidity with blot time of 0.5 s and blot force of 0 using a Mark IV Vitrobot (ThermoFisher Scientific), followed by flash-frozen in liquid ethane. Cryo-EM images were collected on a 300 kV Titan Krios G3 electron microscope (FEI) equipped with Gatan K3 summit direct detection camera and a GIF-Quantum energy filter with a slit width of 20 eV. The super-resolution counting mode of SerialEM program^45^ was used to capture movies automatically with a pixel size of 1.045. Movie stacks were recorded with the defocus values varying from –0.8 to –1.5 μm and generated by 3 s exposure with 32 frames. The dose rate was 2.1875 electrons per Å^2^ per frame.

A total of 7,659 movies for the MIP-1α–CCR5–G_i1_ complex, 7,565 movies for the RANTES–CCR5–G_i1_ complex, and 6,985 movies for the CCR5–G_i1_ complex were collected and subjected to a beam-induced motion correction using MotionCor2^46^. Gctf software^47^ was used to determine contrast transfer function (CTF) parameters for each image. Guided by a template generated from manual picking, autopicking in RELION-3^48^ was performed to extract particle projections. In total, 7,095,732 particles of the MIP-1α–CCR5–G_i1_ complex, 6,624,475 particles of the RANTES–CCR5–G_i1_ complex, and 5,511,331 particles of the CCR5–G_i1_ complex were extracted for two-dimensional (2D) classification and three-dimensional (3D) classification. For the MIP-1α–CCR5–G_i1_ complex, 1,055,847 particles of the best-looking classes were subjected to 3D auto-refinement and Bayesian polishing using RELION-3. The dataset was then used for focused classification using a mask encompassing MIP-1α. A subset of 745,096 particles was selected for further refinement to yield the final map, which was post-processed in RELION-3 with a *B*-factor of –85 Å^2^. For the RANTES–CCR5–G_i1_ complex, 1,304,062 particles of the best-looking classes were used for 3D auto-refinement and Bayesian polishing. The final map of RANTES–CCR5–G_i1_ was sharpened with a *B*-factor of –100 Å^2^. For the CCR5–G_i1_ complex, the best-looking classes containing 1,531,142 particles were selected to conduct 3D auto-refinement and Bayesian polishing in RELION-3. The final refinement was completed using cryoSPARC^49^, resulting in the final map with a *B*-factor of –60 Å^2^. The reported resolution was determined using gold-standard Fourier Shell Correlation (FSC) with the 0.143 criteria. Local resolution was determined using ResMap^50^.

### Model building and refinement of the G_i1_-bound CCR5 structures

The models of the MIP-1α–CCR5–G_i1_ and RANTES–CCR5–G_i1_ complexes were built using the G_i_ heterotrimer from the μ-opioid receptor (μOR)–G_i_ structure (PDB ID: 6DDE) and the CCR5–MIP-1α crystal structure (this study) or the CCR5–[5P7]RANTES crystal structure (PDB ID: 5UIW) as initial models. The model of the CCR5–G_i1_ complex was built using the G_i_ heterotrimer from the μOR–G_i_ structure and the CCR5–maraviroc crystal structure (PDB ID: 4MBS) as initial models. All the models were docked into the cryo-EM electron density maps using Chimera^51^, followed by iterative manual adjustments in COOT and phenix.real_space_refine in Phenix^52^. The final model of CCR5–G_i1_ contains 276 residues of CCR5 (L33-A90 and F96-Q313). The final model of MIP-1α–CCR5–G_i1_ contains 298 residues of CCR5 (C16-Q313) and 69 residues of MIP-1α (S1-A69). The final model of RANTES–CCR5–G_i1_ contains 277 residues of CCR5 (L33-A92 and G97-Q313) and 50 residues of RANTES (S1-Y14, K25-F41, and R47-L65). The remaining residues of CCR5 and chemokines are disordered and were not modeled. The models were validated using Molprobity^53^. Structural figures were prepared by Chimera or PyMOL (https://pymol.org/2/). The data collection and structure refinement statistics are provided in Table 1.

### Purification of the CCR5–MIP-1α complex

The Cell pellets were thawed on ice and lysed by repeated dounce homogenization and centrifugation in a hypotonic buffer containing 10 mM HEPES, pH 7.5, 10 mM MgCl_2_, 20 mM KCl, and EDTA-free protease inhibitor cocktail (Roche), followed by one wash using a high osmotic buffer containing 10 mM HEPES, pH 7.5, 10 mM MgCl_2_, 20 mM KCl, and 1 M NaCl, and one more wash with the hypotonic buffer to remove the high concentration of NaCl. The purified membranes were resuspended in 10 mM HEPES, pH 7.5, 30% (v/v) glycerol, 10 mM MgCl_2_, 20 mM KCl, and EDTA-free protease inhibitor cocktail, flash-frozen with liquid nitrogen, and stored at –80 °C until further use.

The purified membranes were thawed on ice and solubilized in a buffer containing 50 mM HEPES, pH 7.5, 150 mM NaCl, 0.5% (w/v) *n*-dodecyl-β-D-maltopyranoside (DDM, Anatrace), and 0.1% (w/v) CHS at 4 °C for 3 h. The supernatant was isolated by centrifugation at 160,000 *g* for 30 min and incubated with TALON Superflow Metal Affinity Resin (Clontech) supplemented with 10 mM imidazole at 4 °C overnight. The resin was washed with 20 column volumes of wash buffer containing 25 mM HEPES, pH 7.5, 150 mM NaCl, 0.05% (w/v) DDM, 0.01% (w/v) CHS, 10% (v/v) glycerol, and 20 mM imidazole at 4 °C. The protein was then eluted with 5 column volumes of 25 mM HEPES, pH 7.5, 150 mM NaCl, 10% (v/v) glycerol, 0.05% (w/v) DDM, 0.01% (w/v) CHS, and 300 mM imidazole, and exchanged into 25 mM HEPES, pH 7.5, 150 mM NaCl, 10% (v/v) glycerol, 0.05% (w/v) DDM, 0.01% (w/v) CHS, and 40 mM imidazole using a PD MiniTrap G-25 column (GE Healthcare). The protein was then treated with His-tagged PreScission protease (custom-made) overnight to remove the C-terminal Flag and His tags. The protein was subsequently incubated with Ni-NTA superflow resin (Qiagen) at 4 °C for 1 h to remove the cleaved tags and PreScission protease. The purified complex was concentrated to 30–40 mg ml^–1^ with a 100-kDa molecular weight cut-off concentrator (Millipore) for crystallization trails.

### Crystallization of the CCR5–MIP-1α complex

The CCR5–MIP-1α protein sample was mixed with molten lipid (monoolein and cholesterol 10:1 by mass) at a weight ratio of 1:1.5 (protein: lipid) using a mechanical syringe mixer until a homogenous mesophase was achieved. The mixture was dispensed onto glass sandwich plates (Shanghai FAstal BioTech) in 40 nl drop and overlaid with 800 nl precipitant solution at room temperature using a Gryphon robot (Art-Robbins). Plates were incubated and imaged at 20 °C using an automated incubator-imager (RockImager, Formulatrix). Crystals appeared after 10 days and grew to full size (130 μm × 70 μm × 30 μm) in 70 days in 100 mM HEPES, pH 6.0, 100–250 mM ammonium sulfate, 10–30% (v/v) PEG400, 2–8% (v/v) PPG400. Crystals were collected using 75–100 μm MiTeGen micromounts (M2-L19-75/100, MiTeGen) and immediately flash-frozen in liquid nitrogen.

### X-ray diffraction data collection and structure determination

X-ray diffraction data collection was performed using EIGER16M detector (X-ray wavelength 1.0000 Å) at the SPring-8 BL41XU, Hyogo, Japan. Crystals were exposed with an 11 μm × 9 μm mini-beam for 0.2 s and 0.2° oscillation per frame. Data from 40 best-diffracting crystals were processed by HKL2000^54^. The CCR5–MIP-1α structure was solved by molecular replacement implemented in Phaser-MR^55^ using the structures of CCR5–maraviroc (PDB ID: 4MBS), MIP-1α (PDB ID: 2X69), and rubredoxin (PDB ID: 1IRO) as search models. The correct molecular replacement solution contained one CCR5–MIP-1α complex in the asymmetric unit. Structure refinement was performed using autoBUSTER^56^ and REFMAC5^57^, and manual examination and rebuilding of the model were carried out in COOT^58^ based on both |2*F*_o_ | − |*F*_c_ | and |*F*_o_ | − |*F*_c_ | maps. The Ramachandran plot analysis indicates that 100% of the residues are in favorable (95.67%) or allowed (4.33%) regions (no outliers). The final model of the CCR5–MIP-1α complex contains 303 residues of CCR5 (P8-R223 and E227-Q313), 67 residues of MIP-1α (A3–A69), and 54 residues (M1–E54) of rubredoxin. The remaining N- and C-terminal residues of CCR5 and MIP-1α are disordered and were not modeled.

### Disulfide crosslinking

The 10 × His-tagged CCR5 and Flag-tagged MIP-1α containing respective single cysteine mutations were co-expressed in HEK293F cells (Invitrogen) with tyrosylprotein sulfotransferase 1 (TPST-1) co-transfected. The complex was purified as above described. After protein elution from the TALON resin, the purified sample was analyzed by 10% nonreducing Nu-PAGE and western blot. Western blot was performed to specifically identify the His-tagged receptor and Flag-tagged chemokine. The nitrocellulose membrane was incubated in 10 ml of PBS buffer (137 nM NaCl, 2.7 mM KCl, 10 mM Na_2_HPO_4_, and 2 mM KH_2_PO_4_, pH 7.4), supplemented with 5% milk (w/v) and 5 μl primary antibody (mouse monoclonal anti-poly histidine antibody (Sigma, H1029; 1: 2,000 diluted in PBS) or mouse monoclonal anti-flag M2 antibody (Sigma, F3165; 1: 2,000 diluted in PBS)) for 1 h at room temperature. Then the nonspecifically bound antibody was washed with 10 ml PBST buffer (PBS buffer supplemented with 0.1% Tween (v/v)) for 10 min by 3 times. Followed by incubation with the secondary antibody (goat anti-mouse IgG (Sigma AP124; 1: 2,000 diluted in PBS)) for 1 h at room temperature and three washes with PBST, the crosslinked complex was detected by 5-bromo-4-chloro-3-indolyl-phosphate (BCIP, Sigma).

### Inositol phosphate (IP) accumulation assay

Flag-tagged wild-type and mutant CCR5 receptors were cloned into the expression vector pTT5 (Invitrogen) and expressed in HEK293F cells along with the chimeric Gα protein (Gα_Δ6qi4myr_) at the ratio of plasmids of 2:1 (w/w). Cells were harvested 48 h posttransfection. To measure the cell-surface expression of CCR5, 10 μl cells were mixed with 15 μl Monoclonal ANTI-FLAG M2-FITC antibody (Sigma, F4049; 1:100 diluted by TBS supplemented with 4% BSA). After 20 min reaction, the fluorescence signal on the cell surface was measured by an FCM (flow cytometry) reader (Millipore).

IP1 accumulation was measured using an IP-One Gq assay kit (Cisbio Bioassays, 62IPAPEB) following the manufacturer’s instructions. In brief, the harvested cells were plated in 384-well plates (30,000 cells per well) and treated with different concentrations of MIP-1α or RANTES (1 pM–10 μM diluted in stimulation buffer; see below for expression and purification of the chemokines) at 37 °C for 90 min. Then 3 μl cryptate-labeled anti-IP1 monoclonal antibody and 3 μl d2-labeled IP1, which were pre-diluted in Lysis Buffer (1:20), were added to the wells, and incubated at room temperature for 1 h. The plates were then read by an EnVision multilabel plate reader (PerkinElmer) with excitation at 330 nm and emission at 620 and 665 nm. The values were then converted to IP production by a standard dose-response curve using GraphPad Prism 8.0 (GraphPad Software). The assays were performed in parallel with the measurement of the IP production using the cells only transfected with the receptor as a control. The Gα_Δ6qi4myr_-mediated IP accumulation was calculated by subtracting the portion of control-mediated IP production for the wild-type receptor and all the mutants. EC_50_ and pEC_50_ ± SEM were calculated using nonlinear regression (curve fit) in GraphPad Prism 8.0.

### Cyclic AMP (cAMP) inhibition assay

Inhibition of forskolin-stimulated cAMP production was measured using a LANCE Ultra cAMP Detection kit (PerkinElmer) following the manufacturer’s instructions. Flag-tagged wild-type CCR5 and mutants were cloned into the expression vector pTT5 (Invitrogen) and expressed in HEK293F cells (Invitrogen). Cells were harvested 48 h posttransfection. The cell-surface expression of CCR5 was measured as above described. The harvested cells were plated in 384-well plates (1,000 cells per well) using HBSS buffer (Invitrogen) supplemented with 5 mM HEPES (pH 7.5), 0.1% BSA (Sigma), and 0.5 mM IBMX (Sigma). Cells were stimulated with 2 μM forskolin (Sigma) and different concentrations of MIP-1α or RANTES (0.1 pM–1 μM diluted in stimulation buffer; see below for expression and purification of the chemokines) for 30 min at room temperature. Plates were then treated with the Eu-cAMP tracer and ULight-anti-cAMP working solution for 60 min at room temperature. Fluorescent measurements were acquired by an EnVision multilabel plate reader (PerkinElmer) with excitation at 330 nm and emission at 665 nm. The values were then converted to cAMP production by a standard dose-response curve. EC_50_ and pEC_50_ ± SEM were calculated using GraphPad Prism 8.0.

### Expression and purification of MIP-1α and RANTES

For functional studies, the human MIP-1α and RANTES were respectively cloned into the pFastBac1 vector with an HA signal peptide at the N terminus followed by green fluorescent protein (GFP) to increase the protein expression level and an 8 × His tag at the C terminus. Using the Bac-to-Bac Baculovirus Expression System, MIP-1α or RANTES was expressed in HighFive insect cells (Invitrogen). Cells at a density of 1.5 × 10^6^ cells per ml were infected with high-titre viral stocks. Cells were grown at 27 °C for 48 h. The supernatant was isolated by centrifugation and incubated with Ni-NTA superflow resin (Qiagen) at 4 °C for 1 h after filtration using a 0.22 μm filter (Millipore). The resin was then washed with 20 column volumes of 20 mM HEPES, pH 7.5, 100 mM NaCl, and 20 mM imidazole at 4 °C. The protein was eluted with a buffer containing 20 mM HEPES, pH 7.5, 100 mM NaCl, 10% (v/v) glycerol, and 300 mM imidazole. The protein was concentrated to 40 μM, flash-frozen using liquid nitrogen, and stored at –80 °C until use.

### Molecular dynamics simulations

The atomic coordinates of CCR5–MIP-1α and MIP-1α–CCR5–G_i1_ complexes were extracted from their crystal and cryo-EM structures, respectively, with all residue mutants recovered to the wild-type ones. In the crystal structure, the ICL3-rubredoxin fusion protein was removed and the loop was reconstructed and refined using the cyclic coordinate descent (CCD) and kinematic closure (KIC) protocols in Rosetta V3.10^59,60^, and the missing two N-terminal residues of MIP-1α were repaired as well. In the cryo-EM structure, the missing residues 234–240 in the Gα_i1_ subunit were added by superimposing the cryo-EM structure to the structure of Gα_i_ in complex with rhodopsin (PDB ID: 6CMO) and meanwhile, the missing helical domain (residues 56–181) that fluctuates substantially as indicated by earlier simulations was not included in the simulation model^61,62^. The missing N-terminal residues of CCR5 in the cryo-EM structure (P8–Y15) were repaired according to the conformation of the counterpart in the crystal structure. The protonation states of all titratable residues of the receptor, chemokine, and G_i1_ were evaluated at physiological pH 7.4 using Schrodinger suite software. Then the two processed complex systems were embedded in a bilayer composed of 101 and 176 1-palmitoyl-2-oleoyl-sn-glycero-3-phosphocholine (POPC) lipids using the CHARMM-GUI membrane builder, respectively^63^. Each chemokine–receptor (–G_i1_)–membrane system was solvated in a periodic 0.15 M NaCl TIP3P^64^ water box with a minimum water height of 20.0 Å on top and bottom of the system, giving a total of 98,227 atoms for the CCR5–MIP-1α system and 209,844 atoms for the MIP-1α–CCR5–G_i1_ system. The force fields of Amber ff14SB^65^ and lipid17^66^ were used to model protein and membrane atoms, respectively.

The simulations were then performed using the CPU implementation of the parallelized pmemd program in Amber18 suite of program^67^. Each of the two constructed systems was first energy minimized for 5,000 steps, of which the first 2,500 steps were performed using the steepest descent method and the remaining steps with the conjugate gradient method. Then the system was heated from 0 to 310 K using Langevin dynamics with a constant box volume, with restraints applied on the protein, ligand, and lipid molecules (force constant: 10 kcal/mol/Å^2^). Subsequently, these restraints were gradually removed from lipids in 5 steps, in which the system was equilibrated for 1 ns per step at the constant pressure and temperature ensemble (NPT). Further two-step equilibration was carried out at 310 K for 5 ns with harmonic restraints applied on protein only, starting with a force constant of 5 kcal/mol/Å^2^ on protein backbone and ending with 0.1 kcal/mol/Å^2^ on protein Cα atoms. After that, a 20-ns simulation without any restraints was performed at 310 K and 1 bar in the NPT ensemble. During the simulations, the Particle mesh Ewald (PME) method was used to treat all electrostatic interactions with a cut-off distance of 9 Å^68^. The SHAKE algorithm was used for recording the length of covalent bonds involving hydrogen with an integration time step of 2 fs, and periodic boundary conditions were used to avoid edge effects^69^.

Finally, the Gaussian accelerated molecular dynamics (GaMD) simulations, a sophisticated enhanced sampling MD method that efficiently reduces the system energy barriers so as to achieve accelerated simulation as compared to the conventional MD (cMD)^26,27,70^, were performed on the equilibrated systems using the GaMD module implemented in the GPU version of Amer18. To do that, a 10-ns short cMD simulation was used to collect the potential statistics to define GaMD acceleration parameter values, a 4-ns equilibration was for adding the boost potential, and finally a total of 3 independent GaMD production trajectories were run with randomized initial atomic velocities. All GaMD simulations were run at the “dual-boost” level by setting the reference energy to the lower bound, one boost potential being applied to the total potential and the other to the dihedral energetic term. The average and standard deviation (SD) of the system potential energies were calculated every 400,000 steps (0.8 ns) for the CCR5–MIP-1α system and every 800,000 steps (1.6 ns) for the MIP-1α–CCR5–G_i1_ system, respectively. The upper limit of the boost potential SD was set to 6.0 kcal mol^–1^ for both the dihedral and total potential energetic terms. The coordinates were saved every 1,000 steps for data analysis. The free energy surface (FES) on specific collective variables was calculated with the accompanied reweighting algorithm of GaMD.

### Reporting summary

Further information on research design is available in the Nature Research Reporting Summary linked to this article.

## Supplementary information

Supplementary Information

Reporting Summary

## Data Availability

Atomic coordinates and the cryo-EM density maps for the structures of MIP-1α–CCR5–G_i1_, RANTES–CCR5–G_i1_, and CCR5–G_i1_ have been deposited in the RCSB Protein Data Bank (PDB) under accession codes 7F1Q [10.2210/pdb7F1Q/pdb], 7F1R [10.2210/pdb7F1R/pdb], and 7F1S [10.2210/pdb7F1S/pdb], and the Electron Microscopy Data Bank (EMDB) under accession codes EMD-31422, EMD-31423, and EMD-31424. Atomic coordinates and structure factor files for the CCR5–MIP-1α crystal structure have been deposited in the PDB under accession code 7F1T [10.2210/pdb7F1T/pdb]. All relevant data are available from the corresponding authors upon reasonable request. Source data are provided with this paper. The database used in this study includes PDB 1IRO [10.2210/pdb1IRO/pdb], 2X69 [10.2210/pdb2X69/pdb], 4MBS [10.2210/pdb4MBS/pdb], 4RWS [10.2210/pdb4RWS/pdb], 4XT3 [10.2210/pdb4XT3/pdb], 5UIW [10.2210/pdb5UIW/pdb], 6CMO [10.2210/pdb6CMO/pdb], 6WWZ [10.2210/pdb6WWZ/pdb], 6LFO [10.2210/pdb6LFO/pdb], 6DDE [10.2210/pdb6DDE/pdb], 6D9H [10.2210/pdb6D9H/pdb], 6N4B [10.2210/pdb6N4B/pdb], and 6OIK [10.2210/pdb6OIK/pdb]. Source data are provided with this paper.

## Source data

Source Data
