# Supplementary material for: Structural basis for chemokine recognition and receptor activation of chemokine receptor CCR5
Source: Nat Commun. 2021 Jul 6;12:4151. doi: 10.1038/s41467-021-24438-5 (PMC8260604; doi:10.1038/s41467-021-24438-5)
Supplement: Supplementary file 1 — Supplementary Information [file 41467_2021_24438_MOESM1_ESM.pdf]

Supplementary Information

for

Structural basis for chemokine recognition and receptor activation of chemokine receptor CCR5

Hui Zhang, Kun Chen, et al.

a

Construct information

Construct	Mutation	Truncation	Linker between MIP-1α/ RANTES and CCR5	Disulfide crosslinking between CCR5 and MIP-1α/RANTES
Construct 1	G163N	ΔF320-L352	/	/
Construct 2	G163N	ΔF320-L352	12×GS	CCR5 (T16C)-MIP-1α (T15C)
Construct 3	G163N	ΔF320-L352	12×GS	CCR5 (E172C)-RANTES (F28C)
Construct 4	C58Y, M64A, G163N, A233D, R274A, T284A, K303E	ΔF320-L352	12×GS	CCR5 (T16C)-MIP-1α (T15C)

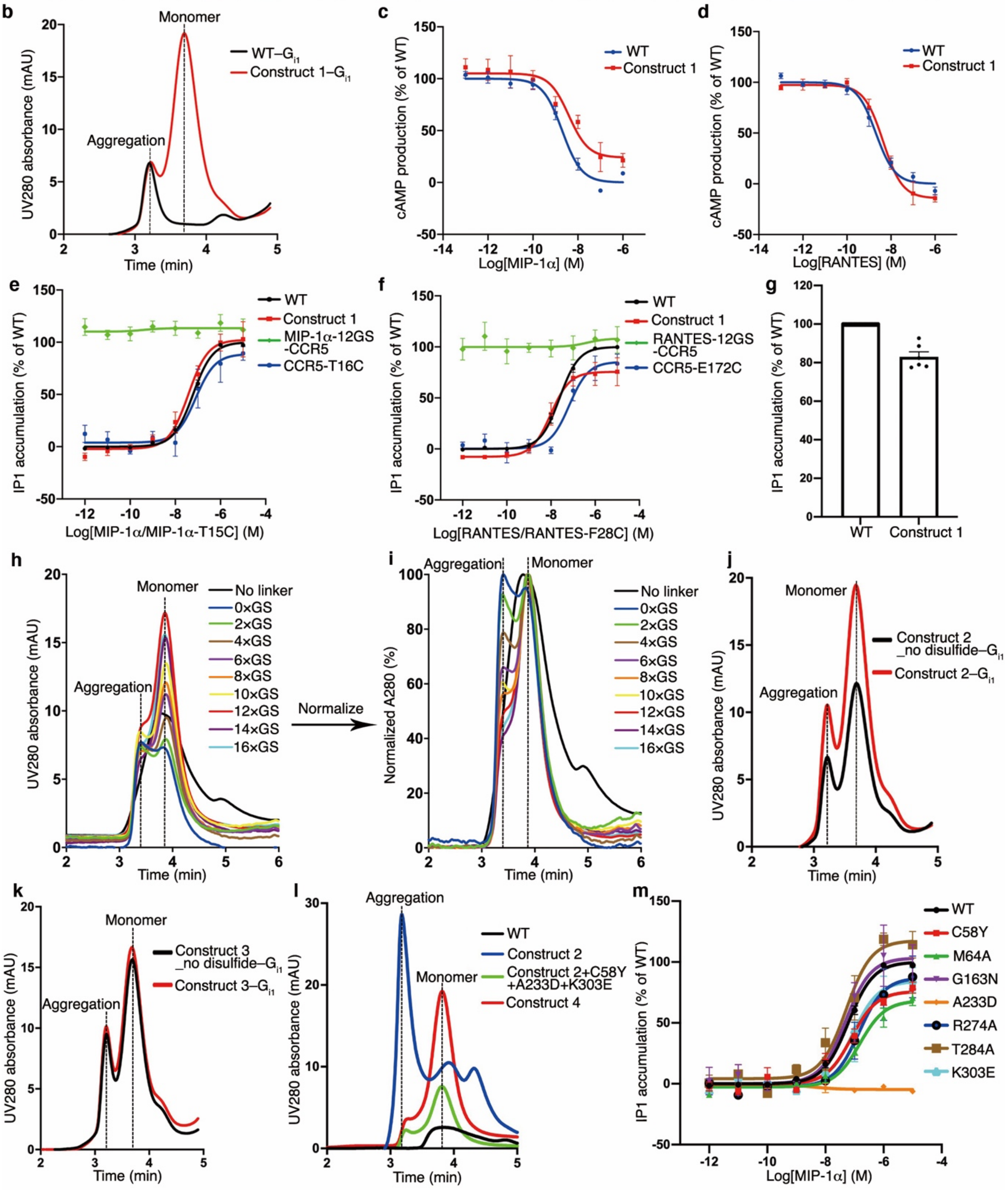

Supplementary Figure 1. Optimization and characterization of CCR5 constructs. **a**, Modifications of constructs used for structure determination. Construct 1, the CCR5 construct used to determine the CCR5–G_{i1} structure; construct 2, the CCR5 construct used to determine the MIP-1α–CCR5–G_{i1} structure; construct 3, the CCR5 construct used to determine the

RANTES–CCR5–G_{i1} structure; construct 4, the CCR5 construct used to determine the CCR5–MIP-1α crystal structure. **b**, Analytical size-exclusion chromatography (aSEC) of wild-type CCR5 (WT) and construct 1 in complex with G_{i1}. The receptor in monomeric and aggregational states are indicated by two dashed lines. The data show that the mutation and truncation of CCR5 improved protein yield and homogeneity of the G_{i1}-bound complex. **c**, **d**, Chemokine-induced cAMP inhibition assays of CCR5. **c**, MIP-1α-induced cAMP inhibition of WT and construct 1. Data are shown as mean ± SEM from twelve (WT) or three (construct 1) independent experiments performed in technical triplicate. **d**, RANTES-induced cAMP inhibition of WT and construct 1. Data are shown as mean ± SEM from ten (WT) or three (construct 1) independent experiments performed in technical triplicate. **e**, **f**, Chemokine-induced IP accumulation assays of CCR5. **e**, MIP-1α-induced IP production of WT, construct 1, and the linked complex MIP-1α-12 × GS-CCR5 (black, red, and green) as well as the IP production of the CCR5 mutant T16C induced by the MIP-1α mutant T15C (blue). **f**, RANTES-induced IP production of WT, construct 1, and the linked complex RANTES-12 × GS-CCR5 (black, red, and green) as well as the IP production of the CCR5 mutant E172C induced by the RANTES mutant F28C (blue). Data are shown as mean ± SEM from at least three independent experiments performed in technical triplicate and are normalized to the surface expression of each protein. See Supplementary Table 1 for detailed statistical evaluation and expression level. The data show that the linked complexes displayed a sustained activity at a similar level to the maximum response of the WT, indicating that the receptor was in a fully chemokine-occupied state. **g**, Basal activity of WT and construct 1 measured by the IP accumulation assay. The data are shown as mean ± SEM from at least six independent experiments performed in technical triplicate. See Supplementary Table 1 for detailed statistical evaluation and expression level. **h, i**, Comparison of the CCR5–MIP-1α complexes with different linkers. **h**, Raw data of aSEC. **i**, Nomalized data of aSEC. The data show that the complex containing the 12 × GS linker has higher protein yield and homogeneity compared to those of the unlinked complex and complexes containing linkers with other lengths. **j, k**, Comparison of the chemokine–CCR5–G_{i1} complexes with or without the designed disulfide bond. **j**, aSEC of the MIP-1α–CCR5–G_{i1} complexes with or without the disulfide bond T16C (CCR5)-T15C (MIP-1α). Construct 2_no disulfide, construct 2 with the disulfide bond T16C (CCR5)-T15C (MIP-1α) removed. **k**, aSEC of the RANTES–CCR5–G_{i1} complexes with or without the disulfide bond E172C (CCR5)-F28C (RANTES). Construct 3_no disulfide, construct 3 with the disulfide bond E172C (CCR5)-F28C (RANTES) removed. **l**, Characterization of the CCR5 construct used for crystallization. The aSEC data show that the fusion and mutations greatly improved protein yield and homogeneity of the CCR5–MIP-1α complex. **m**, MIP-1α-induced IP accumulation assay of CCR5 mutants. Data are shown as mean ± SEM from at least three independent experiments performed in technical triplicate. The assays were performed in parallel with the measurement of the IP production using the cells only transfected with the receptor as a control. The $G\alpha_{\Delta 6qi4myr}$-mediated IP accumulation was calculated by subtracting the portion of control-mediated IP production for the WT receptor and all the mutants. See Supplementary Table 1 for detailed statistical evaluation and expression level. Source data for panels **b-m** are provided as a Source Data file.

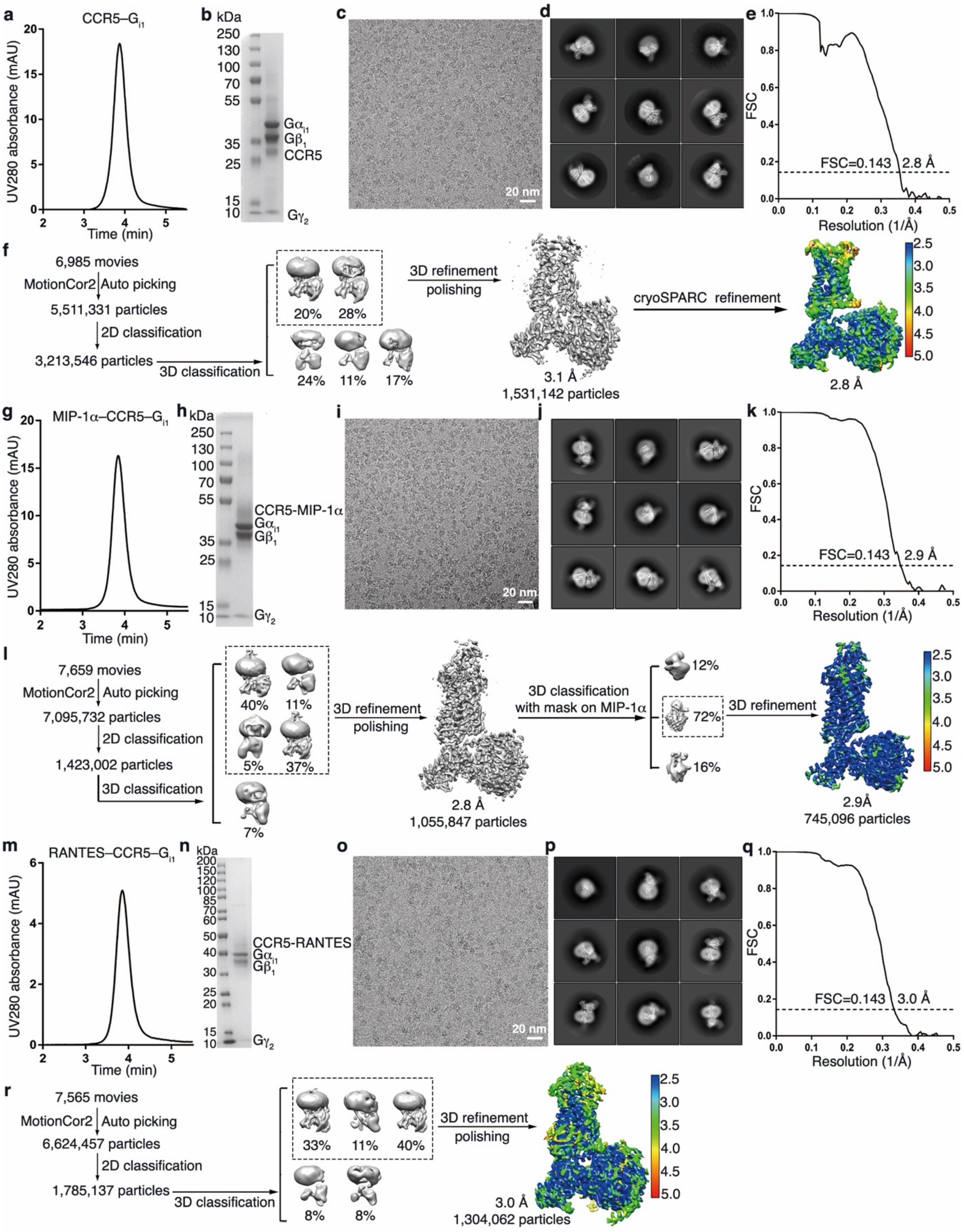

Supplementary Figure 2. Sample preparation and cryo-EM data processing of G_{i1}-bound CCR5 complexes. a-f, Results of the CCR5–G_{i1} complex. **a**, Analytical size-exclusion chromatography (aSEC) of the purified complex. **b**, SDS-PAGE/Coomassie blue stain of the purified complex. Six independent experiments were performed with similar results. Results from a representative experiment are shown. **c**, Representative cryo-EM image from two independent experiments with similar results. **d**, 2D averages. **e**, Gold-standard FSC curve showing an overall resolution at 2.8 Å. **f**, Workflow of cryo-EM data processing with cryo-EM

map colored according to local resolution (Å). **g-l**, Results of the MIP-1α–CCR5–G_{i1} complex. **g**, aSEC of the purified complex. **h**, SDS-PAGE/Coomassie blue stain of the purified complex. Three independent experiments were performed with similar results. Results from a representative experiment are shown. **i**, Representative cryo-EM image from three independent experiments with similar results. **j**, 2D averages. **k**, Gold-standard FSC curve showing an overall resolution at 2.9 Å. **l**, Workflow of cryo-EM data processing with cryo-EM map colored according to local resolution (Å). **m-r,** Results of the RANTES–CCR5–G_{i1} complex. **m**, aSEC of the purified complex. **n**, SDS-PAGE/Coomassie blue stain of the purified complex. Three independent experiments were performed with similar results. Results from a representative experiment are shown. **o**, Representative cryo-EM image from three independent experiments with similar results. **p**, 2D averages. **q**, Gold-standard FSC curve showing an overall resolution at 3.0 Å. **r**, Workflow of cryo-EM data processing with cryo-EM map colored according to local resolution (Å). Source data for panels **a**, **b**, **e**, **g**, **h**, **k**, **m**, **n** and **q** are provided as a Source Data file.

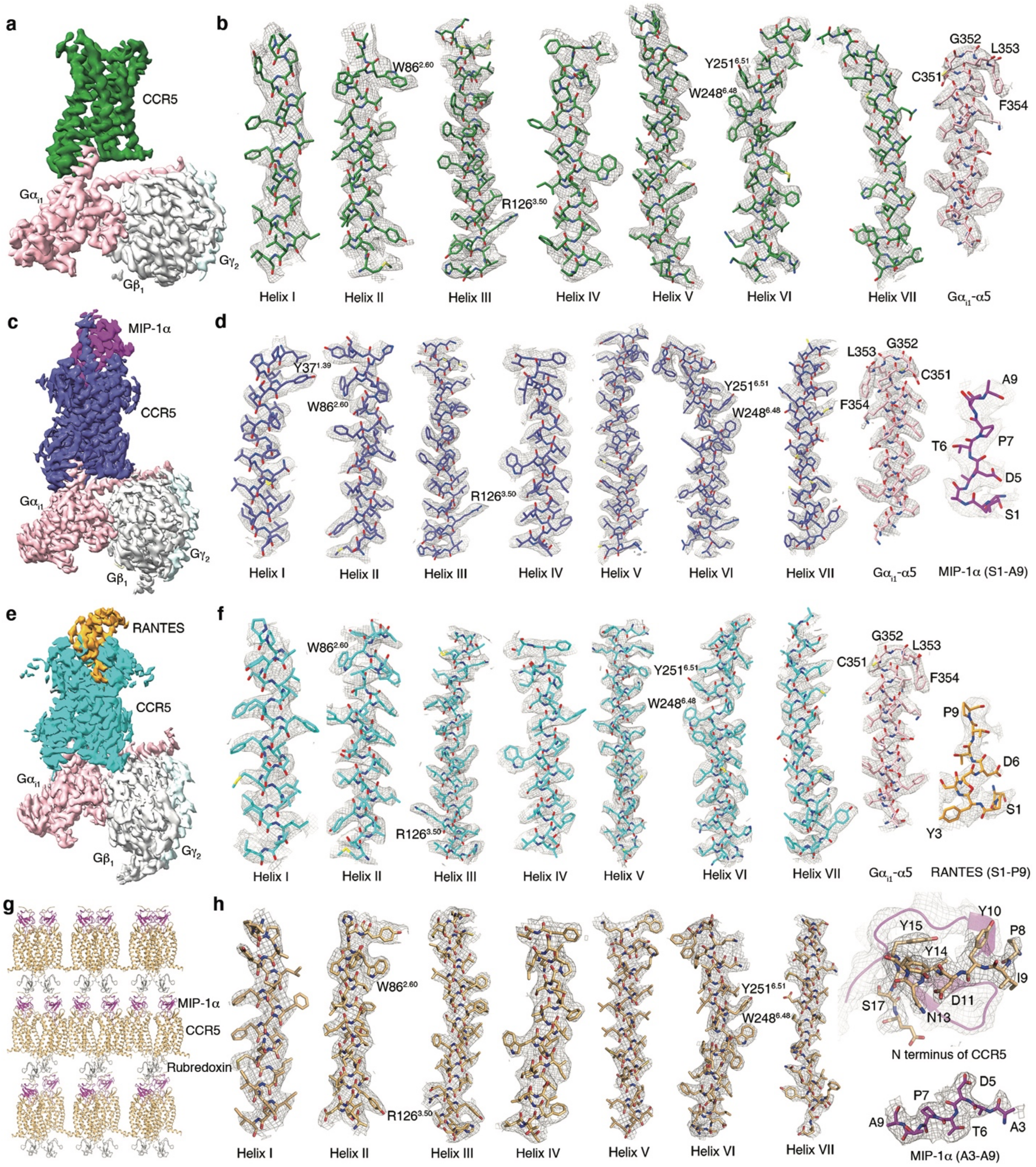

Supplementary Figure 3. Electron density maps of the CCR5 structures. a, Overall cryo-EM map of the CCR5–G_{i1} complex, colored according to chains. **b**, Cryo-EM density map and model of the CCR5–G_{i1} complex structure are displayed for all receptor transmembrane helices and $G\alpha_{i1}$ α5-helix. The structure model is shown as sticks with green (CCR5) and light pink ($G\alpha_{i1}$) carbons. **c**, Overall cryo-EM map of the MIP-1α–CCR5–G_{i1} complex, colored according to chains. **d**, Cryo-EM density map and model of the MIP-1α–CCR5–G_{i1} complex structure are displayed for all receptor transmembrane helices, $G\alpha_{i1}$ α5-helix, and the N terminus of MIP-1α (S1-A9). The structure model is shown as sticks with blue (CCR5), light pink ($G\alpha_{i1}$), and magenta (MIP-1α) carbons. **e**, Overall cryo-EM map of the RANTES–CCR5–G_{i1} complex, colored according to chains. **f**, Cryo-EM density map and model of the RANTES–CCR5–G_{i1} complex structure are displayed for all receptor transmembrane helices, $G\alpha_{i1}$ α5-

helix, and the N terminus of RANTES (S1-P9). The structure model is shown as sticks with cyan (CCR5), light pink ($G\alpha_{i1}$), and orange (RANTES) carbons. **g**, Crystal packing of the CCR5–MIP-1α crystal structure. The structure is shown in cartoon representation and colored gold (CCR5), magenta (MIP-1α), and gray (ICL3-rubredoxin fusion). **h**, Electron densities and model of the CCR5–MIP-1α complex structure are displayed for all receptor transmembrane helices, the N terminus of CCR5 (P8-E18), and the N terminus of MIP-1α (A3-A9).

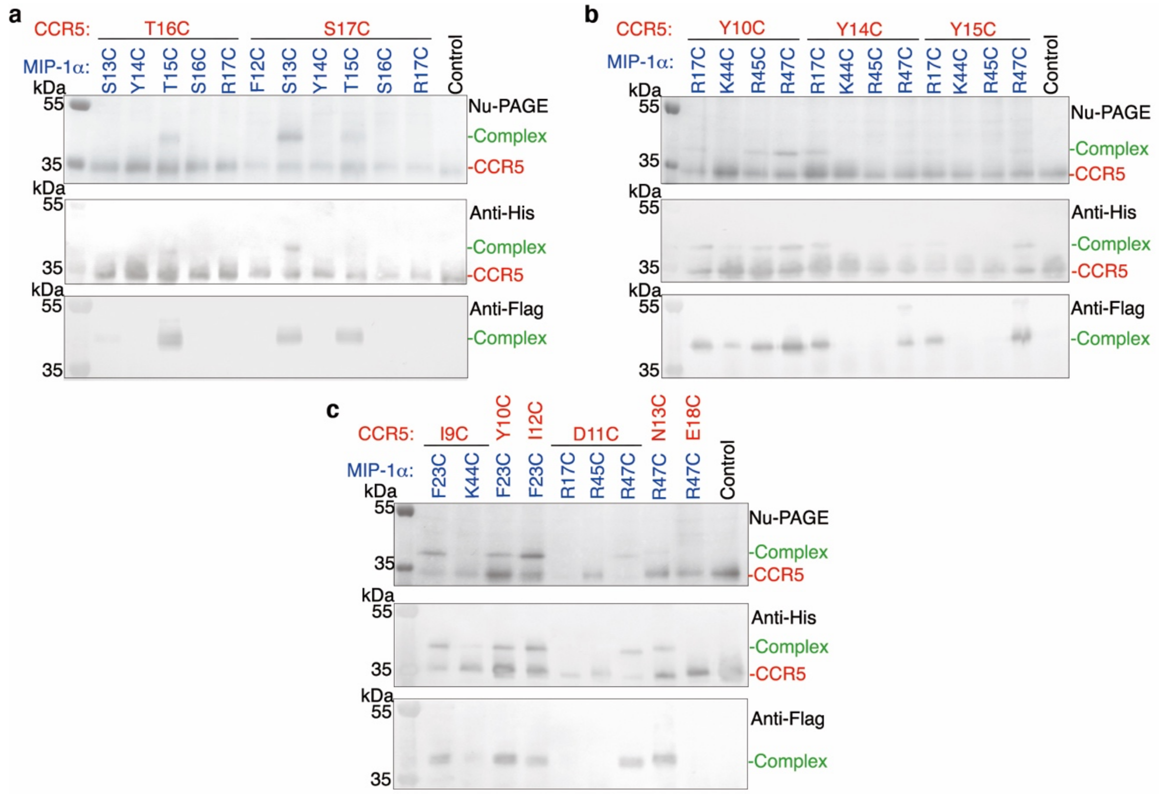

Supplementary Figure 4. Disulfide crosslinking between CCR5 and MIP-1α. a, Crosslinking for the CCR5 mutants T16C and S17C. **b**, Crosslinking for the CCR5 mutants Y10C, Y14C, and Y15C. **c**, Crosslinking for the CCR5 mutants I9C, Y10C, D11C, I12C, N13C, and E18C. The results of 10% non-reducing Nu-PAGE and anti-His/Flag western blot are shown in each panel. The western blot was performed to specifically identify His-tagged CCR5 and Flag-tagged MIP-1α. The disulfide-crosslinked complex can be detected between 35 kDa and 55 kDa by both the anti-His and anti-Flag western blot. The uncrosslinked receptor can only be detected below 35 kDa by the anti-His western blot. The CCR5 co-expressed with MIP-1α without the cysteine mutation pairs was tested in parallel as a control. Four independent experiments were performed with similar results (except for CCR5 (N13C)-MIP-1α (R47C) and CCR5 (E18C)-MIP-1α (R47C) in panel **c**, for which two independent experiments were performed). Results from a representative experiment are shown. Source data are provided as a Source Data file.

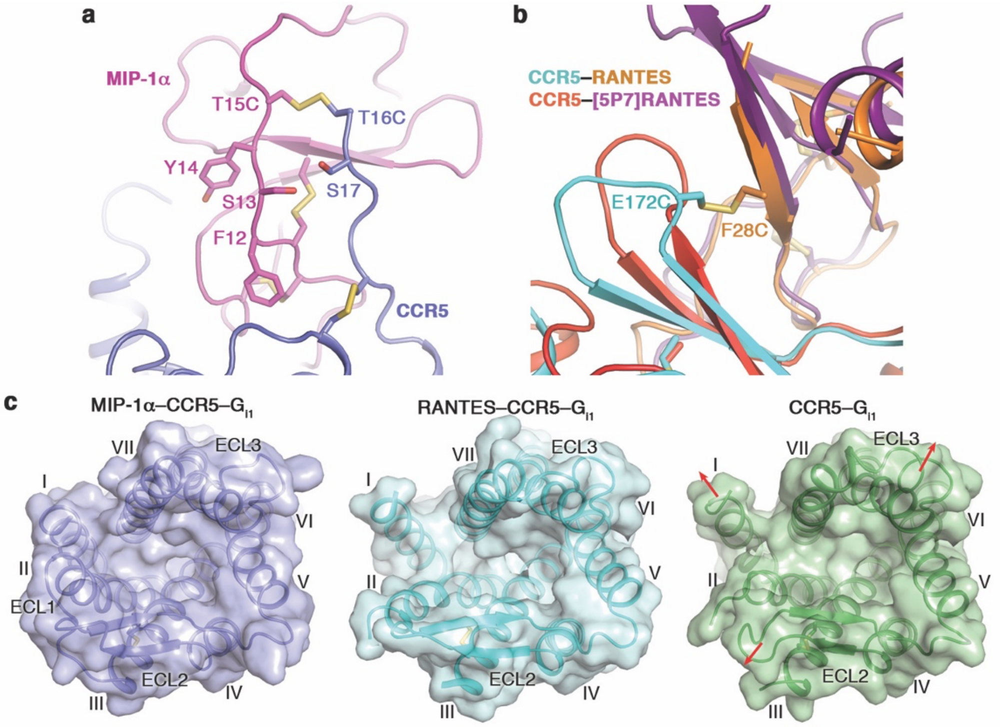

Supplementary Figure 5. Structural comparison of CCR5 structures. a, CCR5 and MIP-1α conformations around the T16C (CCR5)-T15C (MIP-1α) disulfide bond. The MIP-1α–CCR5–G_{i1} structure is shown in cartoon representation and colored blue (CCR5) and magenta (MIP-1α). The CCR5 residue S17 and MIP-1α residues F12-Y14 are shown as sticks. The disulfide bonds are displayed as yellow sticks. **b**, CCR5 and RANTES conformations around the E172C (CCR5)-F28C (RANTES) disulfide bond. The RANTES–CCR5–G_{i1} and CCR5–[5P7]RANTES (PDB ID: 5UIW) structures are shown in cartoon representation. The receptors in the two structures are colored cyan and red, respectively. The chemokines RANTES and [5P7]RANTES are colored orange and purple, respectively. The disulfide bonds are shown as yellow sticks. **c**, Comparison of the ligand-binding pockets in the structures of MIP-1α–CCR5–G_{i1}, RANTES–CCR5–G_{i1}, and CCR5–G_{i1}. The receptors in the three structures are shown in cartoon and surface representations and colored blue, cyan, and green, respectively. The red arrows indicate the outward movements of helix I, ECL2, and ECL3 in the CCR5–G_{i1} structure relative to those in the two chemokine-bound structures.

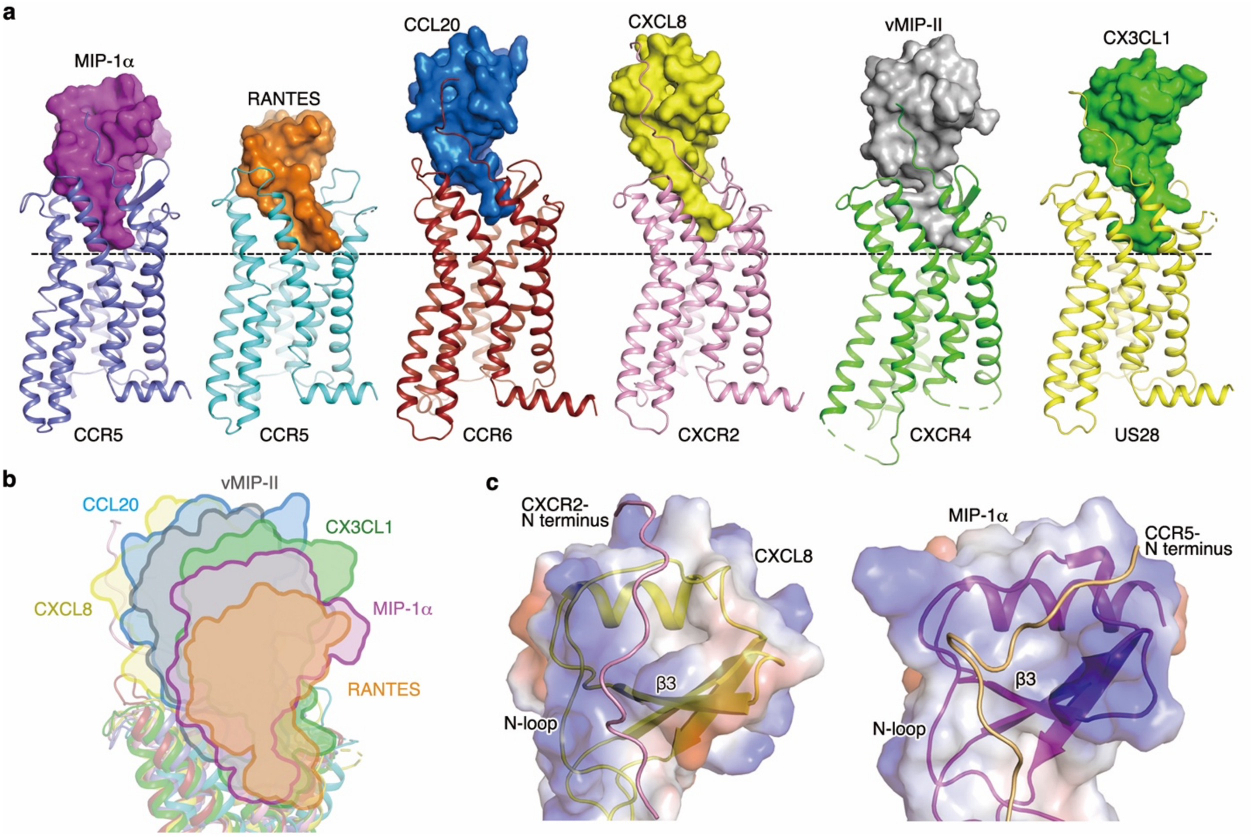

Supplementary Figure 6. Comparison of the chemokine-bound CCR5 structures and other CKR structures. **a**, Comparison of the chemokine-binding sites in the chemokine-bound CKR structures. The receptors and chemokines in the MIP-1α–CCR5–G_{i1} and RANTES–CCR5–G_{i1} structures and the structures of CCL20–CCR6–G_i, CXCL8–CXCR2–G_i, CXCR4–vMIP-II, and US28–CX3CL1 (PDB IDs: 6WWZ, 6LFO, 4RWS, and 4XT3) are shown in cartoon and surface representation, respectively. The deepest position of the chemokines in the CCR5 structures is indicated by a black dashed line. **b**, Comparison of the chemokine binding poses in the chemokine-bound CKR structures. The CKR-chemokine complex structures shown in panel **a** are superimposed by the receptor transmembrane helical bundle. The receptors are shown in cartoon representation. The chemokines are shown in surface and contoured for clarity. **c**, Comparison of the interaction mode between the chemokine and receptor N terminus in CCR5 and CXCR2. The crystal structure of CCR5–MIP-1α and the cryo-EM structure of CXCL8–CXCR2–G_i are shown in cartoon representation. The chemokines in the two structures are also shown as surface colored according to the electrostatic potential from red (negative) to blue (positive).

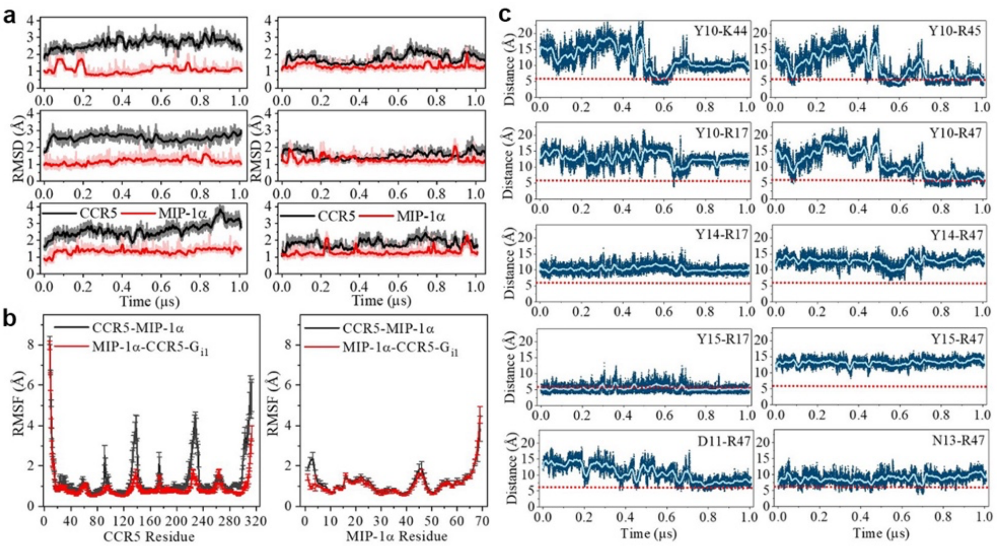

Supplementary Figure 7. GaMD simulation results. a, Time dependence of the root-mean-square deviation (RMSD) of the global structures of CCR5 and MIP-1α during three independent GaMD simulations of CCR5–MIP-1α (left) and MIP-1α–CCR5–G_{i1} (right) complexes. **b**, Residue root-mean-square fluctuations (RMSFs) of CCR5 and MIP-1α in the CCR5–MIP-1α (black) and MIP-1α–CCR5–G_{i1} (red) complexes. Source data are provided as a Source Data file. **c**, Time dependence of β-carbon distances between the specific CCR5 and MIP-1α residues involved in disulfide crosslinking experiments in a representative MD trajectory. Red dashed lines represent the cutoff value (5.5 Å) to evaluate the formation of disulfide bond when these residues are mutated to cysteines.

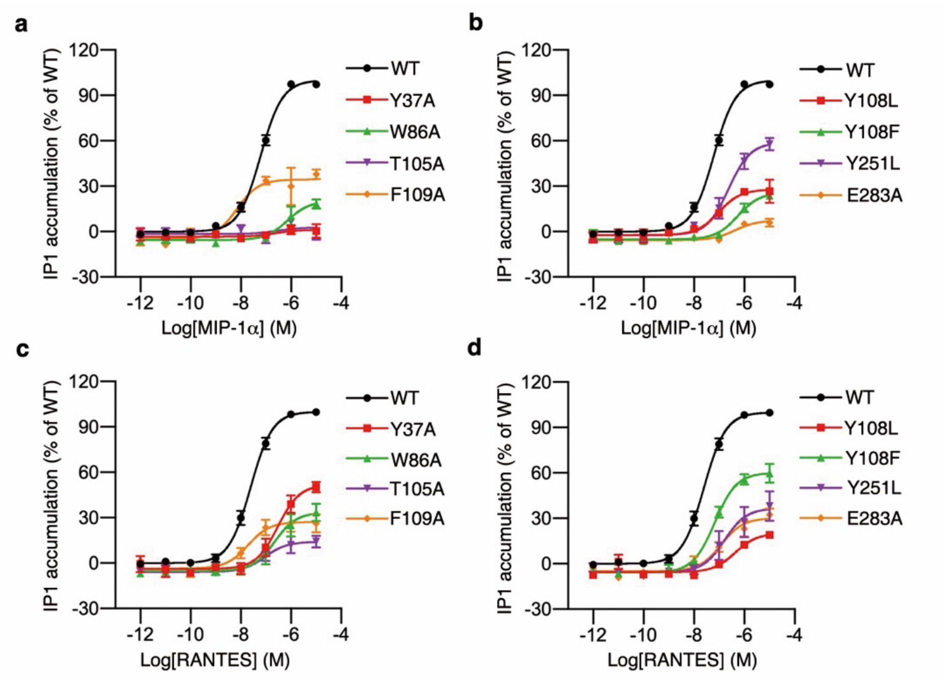

Supplementary Figure 8. IP accumulation assays of wild-type (WT) and mutant CCR5s. Data are shown as mean ± SEM from at least three independent experiments performed in technical triplicate. **a**, **b**, MIP-1α-induced IP accumulation assay using the chimeric Gα protein $G\alpha_{\Delta 6qi4myr}$. **c**, **d**, RANTES-induced IP accumulation assay using the chimeric Gα protein $G\alpha_{\Delta 6qi4myr}$. The assays were performed in parallel with the measurement of the IP production using the cells only transfected with the receptor as a control. The $G\alpha_{\Delta 6qi4myr}$-mediated IP accumulation was calculated by subtracting the portion of control-mediated IP production for the WT receptor and all the mutants. See Supplementary Table 1 for detailed statistical evaluation and expression level. Source data are provided as a Source Data file.

Supplementary Table 1. IP accumulation assays of wild-type (WT) CCR5 and mutants using the chimeric Gα protein $G\alpha_{\Delta 6qi4myr}$

MIP-1α-induced IP accumulation

Mutant[a]	EC_{50} (nM)	Ratio[b]	pEC_{50} ± SEM[c]	Span[c,d] (% of WT)	n[e]	Expression[f] (% of WT)
WT	61	1	7.22 ± 0.04	100 ± 2	34	100
Construct 1[g,h]	38	0.6	7.42 ± 0.16	104 ± 7	3	109 ± 7
MIP-1α-12×GS -CCR5[h]	nd	nd	nd	nd	4	183 ± 21
C58$^{1.60}$Y[i]	79	1	7.10 ± 0.14	76 ± 5*	3	91 ± 14
M64$^{2.38}$A	151	3	6.82 ± 0.13	71 ± 4**	3	116 ± 11
G163$^{4.60}$N	50	0.5	7.31 ± 0.23	103 ± 10	3	125 ± 11
A233$^{6.33}$D	nd	nd	nd	nd	4	113 ± 13
R274$^{7.30}$A	143	2	6.84 ± 0.23	90 ± 10	3	100 ± 16
T284$^{7.40}$A	52	1	7.29 ± 0.17	113 ± 8	3	123 ± 3
K303E	92	2	7.04 ± 0.24	85 ± 9	3	120 ± 12
Y37$^{1.39}$A	nd	nd	nd	nd	4	106 ± 6
W86$^{2.60}$A	724	12	6.14 ± 0.26***	26 ± 4***	3	92 ± 6
T105$^{3.29}$A	nd	nd	nd	nd	3	103 ± 9
Y108$^{3.32}$L	97	2	7.02 ± 0.22	30 ± 3***	3	81 ± 4
Y108$^{3.32}$F	552	7	6.26 ± 0.17***	31 ± 3***	4	102 ± 18
F109$^{3.33}$A	6.9	0.1	8.16 ± 0.23**	39 ± 4***	3	111 ± 7
Y251$^{6.51}$L	238	4	6.62 ± 0.13	61 ± 4***	3	77 ± 8
E283$^{7.39}$A	425	7	6.37 ± 0.35***	13 ± 2***	4	88 ± 19

RANTES-induced IP accumulation

Mutant[a]	EC_{50} (nM)	Ratio[b]	pEC_{50} ± SEM[c]	Span[c,d] (% of WT)	n[e]	Expression[f] (% of WT)
WT	24	1	7.61 ± 0.05	100 ± 2	32	100
Construct 1[g,h]	9.7	0.4	8.02 ± 0.16	83 ± 6	3	109 ± 7
RANTES-12×GS -CCR5[h]	nd	nd	nd	nd	4	193 ± 22
Y37$^{1.39}$A	299	13	6.53 ± 0.16***	56 ± 4***	3	106 ± 6
W86$^{2.60}$A	225	10	6.65 ± 0.25***	39 ± 4***	3	92 ± 6
T105$^{3.29}$A	92	4	7.04 ± 0.27	20 ± 2***	3	103 ± 9
Y108$^{3.32}$L	429	18	6.37 ± 0.21***	26 ± 3***	4	81 ± 4
Y108$^{3.32}$F	67	3	7.18 ± 0.09	66 ± 3***	5	102 ± 18
F109$^{3.33}$A	19	1	7.71 ± 0.25	31 ± 3***	3	111 ± 7
Y251$^{6.51}$L	164	7	6.79 ± 0.28**	42 ± 5***	3	77 ± 8
E283$^{7.39}$A	121	5	6.92 ± 0.18*	35 ± 3***	3	88 ± 19

MIP-1α (T15C)-induced IP accumulation

Mutant[a]	EC_{50} (nM)	Ratio[b]	pEC_{50} ± SEM[c]	Span[c,d] (% of WT)	n[e]	Expression[f] (% of WT)
T16C[h]	80	1	7.10 ± 0.25	85 ± 10	3	65 ± 8

RANTES (F28C)-induced IP accumulation

Mutant[a]	EC_{50} (nM)	Ratio[b]	pEC_{50} ± SEM[c]	Span[c,d] (% of WT)	n[e]	Expression[f] (% of WT)
E172C[h]	68	3	7.17 ± 0.19	85 ± 7	3	77 ± 7

Basal activity

Mutant	Basal activity (% of WT)[j]	n[e]	Expression[f] (% of WT)
WT	100	28	100
WT-maraviroc	34 ± 3***	7	/
Construct 1[g]	83 ± 3***	6	110 ± 5
W86$^{2.60}$F	69 ± 4***	6	119 ± 2
W86$^{2.60}$A	53 ± 4***	6	92 ± 6

Y108$^{3.32}$F	35 ± 4***	6	102 ± 18
Y251$^{6.51}$F	44 ± 4***	5	70 ± 1***

[a]The chimeric $G\alpha_{\Delta6qi4myr}$-mediated IP accumulation assays were performed in parallel with the measurement of the IP production using the cells only transfected with the receptor as a control. The $G\alpha_{\Delta6qi4myr}$-mediated IP accumulation was calculated by subtracting the portion of control-mediated IP production for the WT and all the mutants.
[b]The EC_{50} ratio, $EC_{50(mutant)}/EC_{50(WT)}$, represents the shift between the WT and mutant curves, and characterizes the effect of the mutations on receptor signaling.
[c]Data are shown as mean ± SEM from at least three independent experiments performed in technical triplicate. *P<0.01; **P<0.001; ***P<0.0001 by one-way ANOVA followed by Dunnett's post-test, compared with the response of the WT.
[d]The span is defined as the window between the maximal chemokine response (E_{max}) and the vehicle (no chemokine). nd (not determined) refers to data where a robust concentration response curve could not be established within the concentration range tested, such that an E_{max} was not reached and therefore span could not be calculated.
[e]Sample size; the number of independent experiments performed in technical triplicate.
[f]Protein expression levels of CCR5 constructs at the cell surface were determined in parallel by flow cytometry with an anti-FLAG antibody and reported as per cent compared to the WT from three independent measurements performed in duplicate.
[g]Construct 1, the CCR5 construct used to determine the CCR5–G_{i1} structure.
[h]Data are normalized to the surface expression level for consistence with Supplementary Fig. 1e, f.
[i]All mutations were introduced in the WT.
[j]The IP accumulation assay was performed in parallel with the measurement of the IP production using the cells only transfected with the chimeric Gα protein $G\alpha_{\Delta6qi4myr}$ as a control. The basal activity was calculated by subtracting the IP production measured in the control for the WT receptor and all the mutants and is shown as the per cent of the WT activity. Data are shown as mean ± SEM from at least five independent experiments performed in technical triplicate. ***P<0.0001 by one-way ANOVA followed by Dunnett's post-test, compared with the basal activity of the WT.

Supplementary Note

Design of disulfide bridge between CCR5 and chemokine

Guided by the previously determined CXCR4–vMIP-II structure[1], five pairs of single cysteine mutations of CCR5 and MIP-1α were designed by grouping the CCR5 mutant T16C with the MIP-1α mutant S13C, Y14C, T15C, S16C, or R17C to reduce flexibility of the receptor N terminus. Among these mutant pairs, T16C (CCR5) showed formation of complex when co-expressed with T15C (MIP-1α) but not with the others (Supplementary Fig. 4a). Such specificity of disulfide formation was also observed for the CCR5 mutant S17C, which displayed the highest proportion of trapped complex when co-expressed with the MIP-1α mutant S13C (Supplementary Fig. 4a). This agrees with our MIP-1α-bound CCR5 structures, where these two residues are in close contact with their side chains facing each other (Supplementary Fig. 5a). These data support the compatibility of the disulfide crosslinking strategy with the native complex geometry. Similar strategy was also applied to the CCR5–RANTES complex, in which the second extracellular loop (ECL2) of CCR5 was crosslinked with the β1-strand of RANTES through a disulfide bridge between E172C (CCR5) and F28C (RANTES). Comparison of our RANTES–CCR5–G_{i1} complex structure and the previously published CCR5–[5P7]RANTES structure[2] revealed a limited alteration of the receptor and ligand conformations around this disulfide bond (Supplementary Fig. 5b), indicating that it has little effect on receptor-chemokine interaction.

Supplementary references

1 Qin, L. *et al.* Crystal structure of the chemokine receptor CXCR4 in complex with a viral chemokine. *Science* **347**, 1117-1122 (2015).

2 Zheng, Y. *et al.* Structure of CC Chemokine Receptor 5 with a Potent Chemokine Antagonist Reveals Mechanisms of Chemokine Recognition and Molecular Mimicry by HIV. *Immunity* **46**, 1005-1017 (2017).
