# Supplementary material for: Structural basis for chemokine recognition and receptor activation of chemokine receptor CCR5
Source: Nat Commun. 2021 Jul 6;12:4151. doi: 10.1038/s41467-021-24438-5 (PMC8260604; doi:10.1038/s41467-021-24438-5)
Supplement: Supplementary file 2 — Reporting Summary [file 41467_2021_24438_MOESM2_ESM.pdf]

nature portfolio

Corresponding author(s): Ya Zhu, Yechun Xu, Qiang Zhao, Beili Wu

Last updated by author(s): Jun 9, 2021

Reporting Summary

Nature Portfolio wishes to improve the reproducibility of the work that we publish. This form provides structure for consistency and transparency in reporting. For further information on Nature Portfolio policies, see our Editorial Policies and the Editorial Policy Checklist.

Statistics

For all statistical analyses, confirm that the following items are present in the figure legend, table legend, main text, or Methods section.

n/a	Confirmed	
☐	☒	The exact sample size (*n*) for each experimental group/condition, given as a discrete number and unit of measurement
☐	☒	A statement on whether measurements were taken from distinct samples or whether the same sample was measured repeatedly
☐	☒	The statistical test(s) used AND whether they are one- or two-sided<br>*Only common tests should be described solely by name; describe more complex techniques in the Methods section.*
☒	☐	A description of all covariates tested
☒	☐	A description of any assumptions or corrections, such as tests of normality and adjustment for multiple comparisons
☐	☒	A full description of the statistical parameters including central tendency (e.g. means) or other basic estimates (e.g. regression coefficient) AND variation (e.g. standard deviation) or associated estimates of uncertainty (e.g. confidence intervals)
☐	☒	For null hypothesis testing, the test statistic (e.g. *F*, *t*, *r*) with confidence intervals, effect sizes, degrees of freedom and *P* value noted<br>*Give P values as exact values whenever suitable.*
☒	☐	For Bayesian analysis, information on the choice of priors and Markov chain Monte Carlo settings
☒	☐	For hierarchical and complex designs, identification of the appropriate level for tests and full reporting of outcomes
☒	☐	Estimates of effect sizes (e.g. Cohen's *d*, Pearson's *r*), indicating how they were calculated

Our web collection on statistics for biologists contains articles on many of the points above.

Software and code

Policy information about availability of computer code

Data collection	Automated data collection on the Titan Krios was performed using serial EM 3.7.
Data analysis	The following softwares were used in cryo-EM data processing, model building, and structure validation: MotionCor2 vl.4.2, Gctf vl.06, RELION-3, cryoSPARC V2.15.0., ResMap 1.1.4, UCSF Chimera 1.15, COOT 0.8.9, PHENIX 1.19.2, and MolProbity 4.2.<br>The following software was used in X-ray diffraction data processing and model building: HKL2000 v717, PHENIX 1.17.1-3660, COOT 0.8.9, and Buster v. 2.8.0 (BUSTER, RhoFit, Gelly, Grade, buster-report and Pipedream).<br>The functional data were analyzed by Graph Pad Prism 8.0.<br>The figures were prepared using PyMOL 1.8 and UCSF Chimera 1.15.

For manuscripts utilizing custom algorithms or software that are central to the research but not yet described in published literature, software must be made available to editors and reviewers. We strongly encourage code deposition in a community repository (e.g. GitHub). See the Nature Portfolio guidelines for submitting code & software for further information.

Data

Policy information about availability of data

All manuscripts must include a data availability statement. This statement should provide the following information, where applicable:

- Accession codes, unique identifiers, or web links for publicly available datasets
- A description of any restrictions on data availability
- For clinical datasets or third party data, please ensure that the statement adheres to our policy

Atomic coordinates and the cryo-EM density maps for the structures of MIP-1a–CCR5–Gi1, RANTES–CCR5–Gi1, and CCR5–Gi1 have been deposited in the RCSB Protein Data Bank (PDB) under accession codes 7F1Q, 7F1R, and 7F1S, and the Electron Microscopy Data Bank (EMDB) under accession codes EMD-31422,

EMD-31423, and EMD-31424. Atomic coordinates and structure factor files for the CCR5–MIP-1a crystal structure have been deposited in the PDB under accession code 7F1T. All relevant data are available from the corresponding authors upon reasonable request. Source data are provided with this paper. The database used in this study includes PDB 1IRO, 2X69, 4MBS, 4RWS, 4XT3, 5UIW, 6CMO, 6WWZ, 6LFO, 6DDE, 6D9H, 6N4B, and 6OIK.

Field-specific reporting

Please select the one below that is the best fit for your research. If you are not sure, read the appropriate sections before making your selection.

☒ Life sciences ☐ Behavioural & social sciences ☐ Ecological, evolutionary & environmental sciences

For a reference copy of the document with all sections, see nature.com/documents/nr-reporting-summary-flat.pdf

Life sciences study design

All studies must disclose on these points even when the disclosure is negative.

Sample size	No statistical methods were used to predetermine sample size. All functional data were obtained from at least three independent experiments to ensure each data point was repeatable and comparable to other published studies. Wild-type receptor was tested in parallel as a control with a large number of repeats. Sample size for the cryo-EM studies was determined by availability of microscope time and to ensure unambiguous modeling of the structures. Due to radiation damage, X-ray diffraction data collection of the protein crystals was limited to 5-10 degree per crystal. To collect a complete data set for structure determination, diffraction data from multiple crystals were integrated and scaled using HKL2000. By calculating completeness of the data set, diffraction data from 40 CCR5-MIP-la crystals were used to ensure the completeness was close to 100%.
Data exclusions	No data were excluded from the analyses.
Replication	All functional assays were performed in technical triplicate and reliably repeated within one month. All attempts at replication were successful.
Randomization	Randomization is not relevant to this study, as all experiments did not allocate experimental groups.
Blinding	Blinding is not relevant to this study, as no subjective allocation was involved in any of the structural and functional experiments.

Reporting for specific materials, systems and methods

We require information from authors about some types of materials, experimental systems and methods used in many studies. Here, indicate whether each material, system or method listed is relevant to your study. If you are not sure if a list item applies to your research, read the appropriate section before selecting a response.

Materials & experimental systems

n/a	Involved in the study	
☐	☒	Antibodies
☐	☒	Eukaryotic cell lines
☒	☐	Palaeontology and archaeology
☒	☐	Animals and other organisms
☒	☐	Human research participants
☒	☐	Clinical data
☒	☐	Dual use research of concern

Methods

n/a	Involved in the study	
☒	☐	ChIP-seq
☒	☐	Flow cytometry
☒	☐	MRI-based neuroimaging

Antibodies

Antibodies used	Cryptate-labelled anti-lPl monoclonal antibody: CisBio Bioassays, Cat#621PAPEC, 1:20 diluted in lysis buffer;<br>Monoclonal Anti-FLAG M2-FITC antibody: Sigma-Aldrich, Cat#F4049, 1:100 diluted in TBS+4% BSA;<br>Monoclonal Anti-polyHistidine antibody: Sigma-Aldrich, Cat# H1029, 1:2,000 diluted in PBS;<br>Mouse monoclonal anti-flag M2 antibody: Sigma-Aldrich, Cat#F3165, 1:2,000 diluted in PBS;<br>Polyclonal Goat Anti-Mouse IgG Antibody, Sigma-Aldrich, Cat# AP124, 1:2,000 diluted in PBS.
Validation	All antibodies were commercially obtained and validation reports are available on the supplier website:<br>Cryptate-labelled anti-lPl monoclonal antibody: https://www.cisbio.cn/ip-one-gq-kit-4045l#section-products-tabs-product;<br>Monoclonal Anti-FLAG M2-FITC antibody: https://www.sigmaaldrich.com/technica1-documents/articles/biofiles/antibodies-to-peptides.html;<br>Monoclonal Anti-polyHistidine antibody: https://www.sigmaaldrich.cn/CN/zh/product/sigma/h1029?context=product;<br>Mouse monoclonal anti-flag M2 antibody: https://www.sigmaaldrich.cn/specification-sheets/332/602/F3165-BULK_________SIGMA_____.pdf;

Polyclonal Goat Anti-Mouse IgG Antibody: https://www.sigmaaldrich.cn/CN/zh/product/mm/ap124?context=product.

Eukaryotic cell lines

Policy information about cell lines

Cell line source(s)	The High Five, Sf9, HEK293F cell lines were originally obtained from Invitrogen.
Authentication	None of the cell lines have been authenticated.
Mycoplasma contamination	The cell lines were negative for mycoplasma contamination.
Commonly misidentified lines (See ICLAC register)	No commonly misidentified cell lines were used.
